# A cell autonomous regulator of neuronal excitability modulates tau in Alzheimer’s disease vulnerable neurons

**DOI:** 10.1093/brain/awae051

**Published:** 2024-03-11

**Authors:** Patricia Rodriguez-Rodriguez, Luis Enrique Arroyo-Garcia, Christina Tsagkogianni, Lechuan Li, Wei Wang, Ákos Végvári, Isabella Salas-Allende, Zakary Plautz, Angel Cedazo-Minguez, Subhash C Sinha, Olga Troyanskaya, Marc Flajolet, Vicky Yao, Jean-Pierre Roussarie

**Affiliations:** Department of Neurobiology Care Sciences and Society, Karolinska Institutet, 17 164, Solna, Sweden; Department of Neurobiology Care Sciences and Society, Karolinska Institutet, 17 164, Solna, Sweden; Department of Neurobiology Care Sciences and Society, Karolinska Institutet, 17 164, Solna, Sweden; Department of Computer Science, Rice University, Houston, TX 77004, USA; Bioinformatics Resource Center, The Rockefeller University, New York, NY 10065, USA; Division of Chemistry I, Department of Medical Biochemistry and Biophysics, Karolinska Institutet, 17 164, Solna, Sweden; Laboratory of Molecular and Cellular Neuroscience, The Rockefeller University, New York, NY 10065, USA; Laboratory of Molecular and Cellular Neuroscience, The Rockefeller University, New York, NY 10065, USA; Department of Neurobiology Care Sciences and Society, Karolinska Institutet, 17 164, Solna, Sweden; Helen and Robert Appel Alzheimer’s Disease Research Institute, Brain and Mind Research Institute, Weill Cornell Medicine, New York, NY 10065, USA; Department of Computer Science, Princeton University, Princeton, NJ 08540, USA; Lewis-Sigler Institute for Integrative Genomics, Princeton University, Princeton, NJ 08544, USA; Center for Computational Biology, Flatiron Institute, Simons Foundation, New York, NY 10010, USA; Laboratory of Molecular and Cellular Neuroscience, The Rockefeller University, New York, NY 10065, USA; Division of Chemistry I, Department of Medical Biochemistry and Biophysics, Karolinska Institutet, 17 164, Solna, Sweden; Department of Anatomy & Neurobiology, Boston University Chobanian and Avedisian School of Medicine, Boston, MA 02118, USA

**Keywords:** Alzheimer, tau pathology, selective vulnerability, immediate early genes, DEK

## Abstract

Neurons from layer II of the entorhinal cortex (ECII) are the first to accumulate tau protein aggregates and degenerate during prodromal Alzheimer’s disease. Gaining insight into the molecular mechanisms underlying this vulnerability will help reveal genes and pathways at play during incipient stages of the disease. Here, we use a data-driven functional genomics approach to model ECII neurons *in silico* and identify the proto-oncogene DEK as a regulator of tau pathology.

We show that epigenetic changes caused by *Dek* silencing alter activity-induced transcription, with major effects on neuronal excitability. This is accompanied by the gradual accumulation of tau in the somatodendritic compartment of mouse ECII neurons *in vivo*, reactivity of surrounding microglia, and microglia-mediated neuron loss. These features are all characteristic of early Alzheimer’s disease.

The existence of a cell-autonomous mechanism linking Alzheimer’s disease pathogenic mechanisms in the precise neuron type where the disease starts provides unique evidence that synaptic homeostasis dysregulation is of central importance in the onset of tau pathology in Alzheimer’s disease.

## Teaser

By modelling neurons from the entorhinal cortex *in silico*, Rodriguez-Rodriguez *et al.* obtain evidence suggesting that the proto-oncogene *DEK* is likely to contribute to the vulnerability of these neurons to Alzheimer’s disease. Reducing DEK levels in these neurons *in vitro* leads to changes reminiscent of early Alzheimer’s disease pathology.

## Introduction

Early stages of neurodegenerative disorders are characterized by the aggregation of ubiquitous proteins in discrete populations of brain cells and the degeneration of these cells. For most diseases this selective vulnerability pattern is unexplained, yet it could yield major insight into pathological mechanisms. Alzheimer’s disease (AD), the world-leading cause of dementia, is defined by the appearance of two hallmark pathological lesions, amyloid plaques [extracellular aggregates of amyloid-β (Aβ) peptides] and neurofibrillary tangles (intracellular aggregates of hyperphosphorylated tau, or NFTs). While plaques are widespread in the neocortex and hippocampus, NFTs follow a well defined regional pattern that is commonly used to stage the disease and that starts in principal neurons from layer II of the entorhinal cortex (ECII) during prodromal AD.^1-3^.

Neuroimaging studies have shown that NFTs are the feature that best correlates with regional cortical atrophy^4^ and clinical symptoms of the disease,^5,6^ suggesting a central role of tau in AD pathogenesis. The mechanisms responsible for the progression of neurodegeneration from the entorhinal cortex (EC) to regions that are affected later in the disease remain to be understood, but the propagation of pathological tau along axonal routes has recently gained attention.^7,8^ Subcortical structures like the locus coeruleus (LC) also display early dysfunction and phosphorylated tau accumulation in ‘pretangles’.^9-11^ However, the LC may present tau seed propagation activity only after the EC.^12,13^ ECII neurons might thus form pathological tau *de novo* at the onset of the disease and could be a reservoir for tau spread to other regions. Understanding why ECII neurons start accumulating tau during the earliest stages of AD could therefore reveal a major intervention point for disease-modifying drugs.

We recently used high quality molecular signatures of ECII neurons, along with a large compendium of genomics data to build maps of functional gene interactions within the context of ECII neurons.^14^ Contrasting ECII neurons with neurons more resistant to AD, we found that pathways related to microtubule remodelling were particularly salient in ECII neurons. To investigate AD processes in the specific context of ECII neurons, we used the NetWAS 2.0 (Network-Wide Association Study 2.0) algorithm, which leveraged our ECII functional map along with genome wide association study data for NFT formation.^14,15^ NetWAS 2.0 reprioritized genes based on their association with tau pathology within vulnerable neurons and identified four gene modules—groups of genes with predicted common involvement in biological pathways—potentially contributing to NFT formation in AD. Of of these four modules, one displayed higher connectivity, i.e. higher predicted functional interaction in common cellular processes, in ECII neurons than in any other neuron type, suggesting it might represent cellular processes that are more central in ECII neurons than in other neurons. Furthermore, genes within this module are involved in axonal plasticity and affected by two main AD drivers: ageing and amyloid pathology.^14^

In the present work, we hypothesized that this module could yield major insight into the cascade cell autonomously leading to tau pathology in ECII neurons in AD and searched for core regulators that perturb it. This led to the identification of the proto-oncogene DEK as a hub gene of this module.

Establishing a roadmap for the identification of neuron-type specific regulatory processes, we first experimentally validate in ECII neurons the predictions of our ECII-specific *in silico* models. We thus demonstrate that DEK is indeed a central regulator of the vulnerability module. We further show that perturbations in this module cause tau protein accumulation and somatic redistribution in EC neurons *in vitro* and *in vivo*. This is accompanied by epigenetic dysregulation of immediate early gene (IEG) induction, pathological alterations in neuronal excitability and microglia reactivity. Taken together, we give here proof-of-principle that our vulnerable neuron-centric systems-level approach can yield novel drivers of tau accumulation, and identify a link between several pathological processes occurring during incipient AD.

## Materials and methods

### Mouse models

ECII-bacTRAP mice were previously generated in our laboratory.^14^ They are transgenic for the BAC #RP23-307B16 where a cDNA encoding eGFP-L10a (enhanced green fluorescent protein fused to the L10a subunit of the ribosome) was integrated before the start codon of Sh3bgrl2. As a result, they express eGFP-L10a under the control of the regulatory regions of the ECII enriched Sh3bgrl2 gene, which leads to specific expression of eGFP-L10a expression in ECII neurons. Human-tau transgenic mice (hMAPT mice)^16^ were obtained from Jackson [B6.Cg-*Mapt*^tm1(EGFP)Klt^ Tg(MAPT)8cPdav/J, Strain #005491]. Experiments were performed in 12–15 month old hMAPT mice and 6–8 month old ECII-bacTRAP mice. The groups were age-matched for each experiment.

Mice were maintained on a 12 h dark/light cycle and provided with food and water *ad libitum*. All the mouse experiments were approved by the Rockefeller University Institutional Animal care and Use Committee (IACUC protocols #16902 and 19067-H) and Karolinska Institutet (4884-2019) ethical committee.

### Mouse primary neuron cultures

Entorhinal cortices from E17 C57Bl/6J mouse embryos (Jackson, stock 000664) were incubated at 37°C in 0.05% trypsin/EDTA (#T11493, ThermoFisher) for 10 min. After centrifugation, the tissue pellet was dissociated in Hank’s balanced salt solution (HBSS) containing 0.5 mg/ml DNAse I (Merck) with a glass Pasteur pipette. Cells were seeded at 50 000 cells/cm^2^ in Neurobasal medium (ThermoFisher), supplemented with 2% B-27 (ThermoFisher) and 2 mM GlutaMAX (ThermoFisher), and grown at 37 °C in a humidified 5% CO_2_-containing atmosphere. All experiments were performed after a minimum of 10 days *in vitro* (DIV).

### AAVs

Purified adeno-associated virus (AAVs) stocks were produced by Vector Biolabs. The viruses used for *Dek* silencing were: AAV1-mCherry-U6-mDEK-shRNA (shRNA sequence: 5'-CCGG-CGAACTCGTGAAGAGGATCTTCTCGAGAAGATCCTCTTCACGAGTTCG-TTTTT −3’) and AAV1-mCherry-U6-scrmb-shRNA (control shRNA sequence: 5’- CCGG-CAACAAGATGAAGAGCACCAACTCGAGTTGGTGCTCTTCATCTTGTTG-TTTTT-3’). The viruses used for *Dek* overexpression were: AAV1-hSyn1-mDEK-IRES-mCherry (expressing the cDNA encoding for mouse DEK—NM_025900) and AAV1-hSyn1-mCherry-WPRE (empty control).

### Entorhinal cortex mouse primary neuron treatments

#### AAVs transduction

EC neurons in primary culture at 7 DIV were transduced by a full media change to media containing either control, mDEK-IRES or mDEK-shRNA AAVs at a concentration of 1 × 10^10^ genome copies per ml of media (GC/ml). Neurons were maintained in virus-containing media until the day of the experiment.

#### KCl stimulation

EC neurons were stimulated by adding either vehicle (PBS) or 50 mM KCl to the culture media. Neurons were incubated with KCl for the whole duration of the experiment.

### Epigenomics analysis

Mass spectrometry analysis of histone post-translational modifications (Mod-spec) and H3K36ac cleavage under targets and tagmentation (CUT&Tag) were performed by Activ Motif on primary EC neurons transduced with control or *Dek*-silencing AAVs (Supplementary material, ‘Methods’ section).

### Electrophysiology

Mouse EC neurons in primary culture were grown on glass coverslips. Coverslips were placed in a submerged chamber perfused with aerated artificial CSF: 124 mM NaCl, 30 mM NaHCO_3_, 10 mM glucose, 1.25 mM NaH_2_PO_4_, 3.5 mM KCl, 1.5 mM MgCl_2_, 1.5 mM CaCl_2_, at 36°C with a perfusion rate of 1–2 ml/min. Coverslips were left undisturbed for 5 min before any recording. Patch-clamp (whole-cell) recordings were performed with borosilicate glass microelectrodes (4–6 MΩ) from the soma of visually identified pyramidal cells using IR-DIC microscopy (Zeiss Axioskop) using a Multiclamp 700B (Molecular Devices). For neuronal electrophysiological properties and spontaneous excitatory postsynaptic current (sEPSC) measurements (Vh = −70 mV) a potassium-based intracellular solution was used: 122.5 mM K-gluconate, 8 mM KCl, 4 mM Na_2_-ATP, 0.3 mM Na_2_-GTP, 10 mM HEPES, 0.2 mM EGTA, 2 mM MgCl_2_, 10 mM Na_2_-Phosphocreatine, set to pH 7.2–7.3 with KOH, osmolarity 270–280 mOsm. The signals were sampled and low pass filtered at 2 kHz, digitized and stored using a Digidata 1440A and pCLAMP 10.4 software (Molecular Devices, CA, USA).

#### Resting membrane potential

Resting membrane potential (RMP) was determined in current-clamp mode after breaking the membrane and calculated from the mean of 1 min recording using Clampfit11.2.

#### Cell capacitance

Cell capacitance (Cm) and Tau (τ) values were taken directly from the Clampex membrane test tool and amplifier readings.

#### Input resistance

Input resistance was measured from subthreshold hyper and depolarizing current steps (−20 to 20 pA, 10 pA increments, 700 ms; Vh = −70 mV) and calculated using Clampfit11.2.

#### Rheobase

First-spike latency measurements were done by applying a rheobase protocol to PCs in current-clamp whole cell configuration to elicit the firing of a single action potential (AP) (10 pA increments, 300 ms). The current at which the first AP was fired was defined as current threshold. First-spike latency was then calculated as the time between the beginning of the test current pulse and the maximum amplitude of the AP.

#### Sag

To measure the absolute difference between the steady state decrease in the voltage and the largest decrease in the voltage following a hyperpolarizing step (sag), a hyperpolarizing current pulse was applied to primary cultures (Vh = −60 mV). Amplitude was calculated from the steady state of the current to the peak.

#### Action potential waveform

Long-lasting current steps to evoke one AP were used to analyse AP properties (10 pA increments, 700 ms). AP parameters [amplitude, firing threshold, half-width, maximum rise slope (MRS), time to MRS, maximum decay slope (MDS) and time to MDS], AP waveform plot and analysis were performed in Clampfit11.2.

#### Spontaneous excitatory postsynaptic currents

sEPSCs were detected off-line using MiniAnalysis software (Synaptosoft, Decatur, GA, USA). Charge transfer, event amplitude and event frequency were analysed using Microsoft Excel (Microsoft Office) and GraphPad Prism with the result representing average values taken over 1 min periods.

### Stereotaxic injections

AAVs were injected in the EC of adult mice using an Angle Two mouse stereotaxic frame with a motorized nanoinjector (Leica). Animals were anaesthetized with xylazine (4.5 mg/kg body weight) and ketamine (90 mg/kg body weight) injected peritoneally. An ophthalmic ointment was applied to the anaesthetized animals to prevent corneal drying during the procedure. AAVs were loaded in a 10 μl syringe (#7653-01, Hamilton) with a 33-gauge needle (#7803-05, Hamilton). AAVs (2 μl, 1 × 10^13^ GC/ml) were injected in the mouse EC (AP: −3.70; ML: −4.65; DV: −4.50) with the nanoinjector tilted −4.05°. The same volume and concentration of control AAVs were injected in the contralateral EC (AP: −3.70; ML: + 4.65; DV: −4.50) with a nanoinjector tilt of +4.05°. The injection rate was 0.4 μl/min. Bacitracin antibiotic gel was applied to the surgery wound, which was sutured with a non-absorbable monofilament. To compensate for fluid loss, warm sterile saline solution was injected intraperitoneally (3% of the body weight) and animals were kept on a heated pad and monitored until complete recover from anaesthesia. In cohorts with injection of *Dek* or control AAVs on contralateral sides, the hemisphere receiving the control AAV was alternated for each mouse to minimize potential confounders. The experimental unit was one EC hemisphere. In cohorts where *Dek* or control AAVs were injected bilaterally in different mice, mice receiving *Dek* or control AAVs were alternated. The experiment unit was one mouse (two pooled EC hemispheres). In all cases, the experimenter processing tissue was blinded to the AAV injected in each hemisphere or mouse. No mouse was excluded from the study after injection. The outcome measures were the number of surviving ECII neurons, the levels of Iba-1 around layer II and the total levels of phospho-tau.

### RNA sequencing

#### Primary culture RNAseq

For RNA sequencing (RNAseq) analysis of EC primary cultures, RNA was extracted 4 days after AAV transduction using the Purelink RNA kit. RNA (200 ng) was used to generate RNAseq libraries using the Truseq RNA sample prep kit. Libraries were sequenced using a NextSeq 500 Illumina sequencer.

#### bacTRAP-RNAseq

Entorhinal cortices from both hemispheres were isolated from individual mice and processed for bacTRAP, as previously described^14,17^ (Supplementary material, ‘Methods’ section).

### PLX5622 treatments

PLX5622 compound was obtained from MedChemExpress. Chow containing PLX5622 at 1200 ppm was manufactured by a trained diet preparation operator at Research Diets Inc in AIN-76A rodent diet. Animals were supplied with AIN-76A diet pellets either alone (control) or containing PLX5622 *ad libitum*. Diet supplementation was initiated 5 days before stereotaxic injections were performed and was maintained for the whole duration of the experiments.

### Mouse tissue processing

#### Analysis of mouse tissue samples by western blot and RT-qPCR

In order to reduce the number of animals used in this study and to have a consistent representation of the region of interest and injected area for both western blot and RT-qPCR analysis, dissected ECs were initially subjected to a fast homogenization in 100 μl RNAse free H_2_O supplemented with protease (ThermoFisher), phosphatase (PhoSTOP, Merck) and RNAse (40 U/ml RNAsin, Promega and 20 U/ml Superasin, ThermoFisher) inhibitors with a cordless motor pestle homogenizer. Immediately after, 45 μl of the homogenate was transferred to an RNAse-free tube, mixed with RNA extraction buffer and cleaned up using the Purelink RNA kit (see below). The remaining volume was lysed in RIPA protein extraction buffer (ThermoFisher).

#### Immunofluorescence analysis

Mice were anaesthetized with nembutal and perfused with PBS followed by 4% paraformaldehyde (PFA). Brains were then dissected and post-fixed in 4% PFA for 1 h. Afterwards they were washed with PBS and cryopreserved by incubation in increasing sucrose concentrations (5%, 15% and 30%). They were then embedded in optical coherence tomography (OCT) compound (TissueTek), cut in 40 μm-thick free-floating horizontal sections with a CM3050 Cryostat (Leica) and further preserved in cryoprotectant solution (50% ethylene glycol, 20% glycerol in PBS) at −20°C until further analysis.

### Western blot

Cells or mouse brain tissue was homogenized in RIPA buffer (ThermoFisher), supplemented with protease (ThermoFisher) and phosphatase (PhoSTOP, Merck) inhibitors. The cell extracts were then subjected to SDS-PAGE and transferred to a nitrocellulose membrane. Membranes were blocked in Intercept (TBS) blocking buffer (Li-Cor) 1 h at room temperature and blotted with the primary antibodies overnight at 4 °C. Antibodies used were AT8 (1:2000, ThermoFisher), p-Tau Th181 (1:1000, Cell Signaling), p-Tau Thr231 (1:1000, NovusBio), Tau (1:1000, Dako and Tau5 1:1000, Millipore), LC3a (1:1000, NovusBio) and Iba1 (1:1000, Wako). Beta actin (1:1000, Cell Signaling) or Gapdh (1:1000, Abcam) were used as loading control. The day after membranes were incubated with IRDye 800CW and 680RD mouse and rabbit secondary antibodies (Li-cor) for 1 h at room temperature. Signal detection was performed with a Oddisey scanning system (Li-Cor). Band intensity quantification was performed with the Image Studio Lite software.

### Immunofluorescence

EC neurons in primary culture were seeded in poly-D-lysine-coated round glass coverslips. After the treatments, they were fixed in 4% PFA for 10 min. Fixed cells were then blocked for 30 min in 1% bovine serum albumin (BSA), 0.1% Triton X-100, PBS (ThermoFisher) and incubated with the primary antibody overnight at 4°C. The antibodies used were AT8 (1:1000, ThermoFisher) and MAP2 (1:1000, Abcam). The day after, the cells were washed with PBS and incubated with the secondary antibody in PBS for 1 h at room temperature. The secondary antibodies used were Goat anti-mouse IgG (H + L) cross-adsorbed Alexa fluor 488 (ThermoFisher) and Goat anti-rabbit IgG (H + L) cross-adsorbed Alexa fluor 594 (ThermoFisher). DAPI was used as nuclear staining. Cells were then washed and mounted with ProLong Gold Antifade Reagent (ThermoFisher).

Free-floating horizontal sections (40 μm-thick) were permeabilized in 0.1% fish gelatin, 2% normal goat serum and 0.1% Triton X-100 in PBS for 30 min at room temperature. They were then stained overnight at 4°C with primary antibodies diluted in the same permeabilization buffer. Primary antibodies used were mCherry (1:1000, Abcam), Iba1 (1:1000, Wako), Reelin (1:500, Millipore), AT180 (1:1000, Novus Bio). The day after, samples were washed in PBS and incubated with secondary antibodies for 1 h at room temperature. The secondary antibodies used were Goat anti-mouse IgG (H + L) cross-adsorbed Alexa fluor 488 (ThermoFisher), Goat anti-chicken IgG (H + L) cross-adsorbed Alexa fluor 594 (ThermoFisher) and Goat anti-rabbit IgG (H + L) cross-adsorbed Alexa fluor 647 (ThermoFisher). After washing with PBS, autofluorescence was quenched with Eliminator reagent (Millipore Sigma) following the manufacturer’s instructions and mounted with ProLong Gold Antifade Reagent (ThermoFisher).

#### Image acquisition

All images were acquired on a Zeiss LSM 510 META laser scanning confocal microscope.

#### Iba1 and AT180 fluorescence intensity quantification in mouse sections

A minimum of three pictures were taken from different sections and from each hemisphere from the same mouse (shDEK and Control transduced). The region of interest was established manually by selecting the ECII and on slides where mCherry positive cells (AAV transduced) could be detected. Iba1 and AT180 fluorescence intensity was determined using the threshold selector to identify positive staining signal.

### RT-qPCR

#### Entorhinal cortex neurons in primary culture

RNA extraction, purification, reverse-transcription and qPCR were performed using the TaqMan Fast Advanced Cells-to-CT kit (ThermoFisher), according to the manufacturer’s instructions.

#### Mouse tissue homogenates

RNA was purified using the PureLink RNA mini kit with in-column DNAse-I digest (Qiagen) following the manufacturer’s instructions (ThermoFisher). RNA was then quantified in a nanodrop and ∼400 ng of RNA was reverse-transcribed using the SuperScript III First-Strand Synthesis System with a 1:1 mix of oligo dT and random hexamers oligonucleotides (ThermoFisher), followed by RNAse H digestion. cDNA was then used to run qPCRs using TaqMan Universal PCR master mix.

FAM-labelled TaqMan assays (ThermoFisher) used were: Mm00662582_m1 (*Dek*), Mm00656724_m1 (*Egr1*), Mm00463644_m1 (*Npas4*), Mm00521992_m1 (*Mapt*), Mm00438354_m1 (*Cx3cr1*), Mm07295529_m1 (*C1qa*), Mm00434455_m1 (*Itgam*), Mm99999057_m1 (*Ccl3*), Mm00449152_m1 (*Tyrobp*), Mm00444701_m1 (*Ccl24*), Mm00446026_m1 (*P2ry12*) and Mm00436767_m1 (*Spp1*). *Gapdh* was used as endogenous control (4352932E). All qPCRs were performed in a QuantStudio 12K-flex machine.

### Network connectivity analysis

To identify genes that are strongly connected to the AD vulnerability module, for each gene *g* in the ECII functional network, we calculated a z-score for vulnerability module connectivity:

$z_{\mathrm{connectivity}}=\frac{{\bar{x}}_{g}-\mu }{\sigma /\sqrt{n}}$ , where ${\bar{x}}_{g}$ is the mean of posterior probabilities between *g* and all genes in the AD vulnerability module, and *n* is the number of genes in the module. μ and σ are the mean and standard deviation, respectively of posterior probabilities of all genes in the network to gene *g*. Thus, high *z_connectivity_* represents stronger connectivity between gene *g* to the AD vulnerability module than expected based on gene *g*’s general connectivity patterns within the ECII network.

### Differential gene expression analysis

Following sequencing, adapter and low quality bases were trimmed by fastp^18^ from the raw sequencing files in FASTQ format. Cleaned reads were aligned to the mm10 reference genome using STAR version 2.7.1a.^19^ After alignment, the reads per kilobase of transcript per million mapped reads (RPKM) for all genes in each sample were calculated with R package edgeR.^20^ To analyse differential gene expression between samples, DESeq2^21^ was used, applying the standard comparison mode between two experimental groups.

### Variant effect prediction

Single nucleotide polymorphisms (SNPs) in linkage disequilibrium (LD) with rs145578678 in populations with European ancestry (based on 1000 Genomes phase 3 data collected from the Finnish populations in Finland, British populations in England and Scotland, Iberian populations in Spain, Toscani populations in Italy, and Utah residents with Northern and Western European ancestry) were obtained from Ensembl (release 110)^22^ and analysed with DeepSEA using the Beluga model (https://hb.flatironinstitute.org/deepsea/, hg38 Genome assembly). Only predictions with *P*-value < 0.05 after Bonferroni correction were considered.

### Mass spectrometry analysis of protein abundance

Protein identification and quantification was performed by liquid chromatography-tandem mass spectrometry by the Proteomics Biomedicum Core Facility at the Karolinska Institutet (Supplementary material, ‘Methods’ section).

### Statistical analysis

All statistical analyses were performed with GraphPad Prism 10. Statistical details can be found in the figure legends. In the figures throughout the manuscript, we use the usual *P*-value convention: *0.01 < *P*-value ≤ 0.05; **0.001 < *P*-value ≤ 0.01; ***0.0001 < *P*-value ≤ 0.001; ****0.0001 ≤ *P*-value.

## Results

### Data-driven identification of a tau pathology driver in ECII neurons

To determine drivers of tau pathology in ECII neurons, we honed in on the AD vulnerability module identified by the aforementioned NetWAS 2.0 approach.^14^ We calculated the connectivity of every gene in the ECII functional network to genes in the AD vulnerability module (Supplementary Table 1). Intuitively, high connectivity to genes in the AD vulnerability module indicates a strong functional relationship as a potential regulator of this module. The gene with the highest connectivity to this module was *DEK*, suggesting that it might be a core regulator of tau pathology within vulnerable neurons. Moreover, in the ECII neuron functional network, *DEK* was also predicted to be one of the closest functional interaction partners of *MAPT*, the gene that codes for tau (connectivity score 0.976, ranked 20 out of 23 950; data publicly available at alz.princeton.edu) (Supplementary Table 1), further indicating that DEK might be key to ECII vulnerability.

*DEK* codes for the proto-oncogene DEK, a nuclear phosphoprotein that was identified for the first time in patients with acute myeloid leukaemia^23^ and is frequently upregulated in solid tumours.^24^ It has also been associated with several autoimmune diseases.^25^ DEK is ubiquitously expressed in the CNS (Supplementary Fig. 1), although its role there remains largely unknown. We therefore aimed to characterize DEK function in ECII neurons, and its potential contribution to pathological processes leading to AD.

### DEK regulates vulnerability-associated gene module in the entorhinal cortex

**Figure 1:**
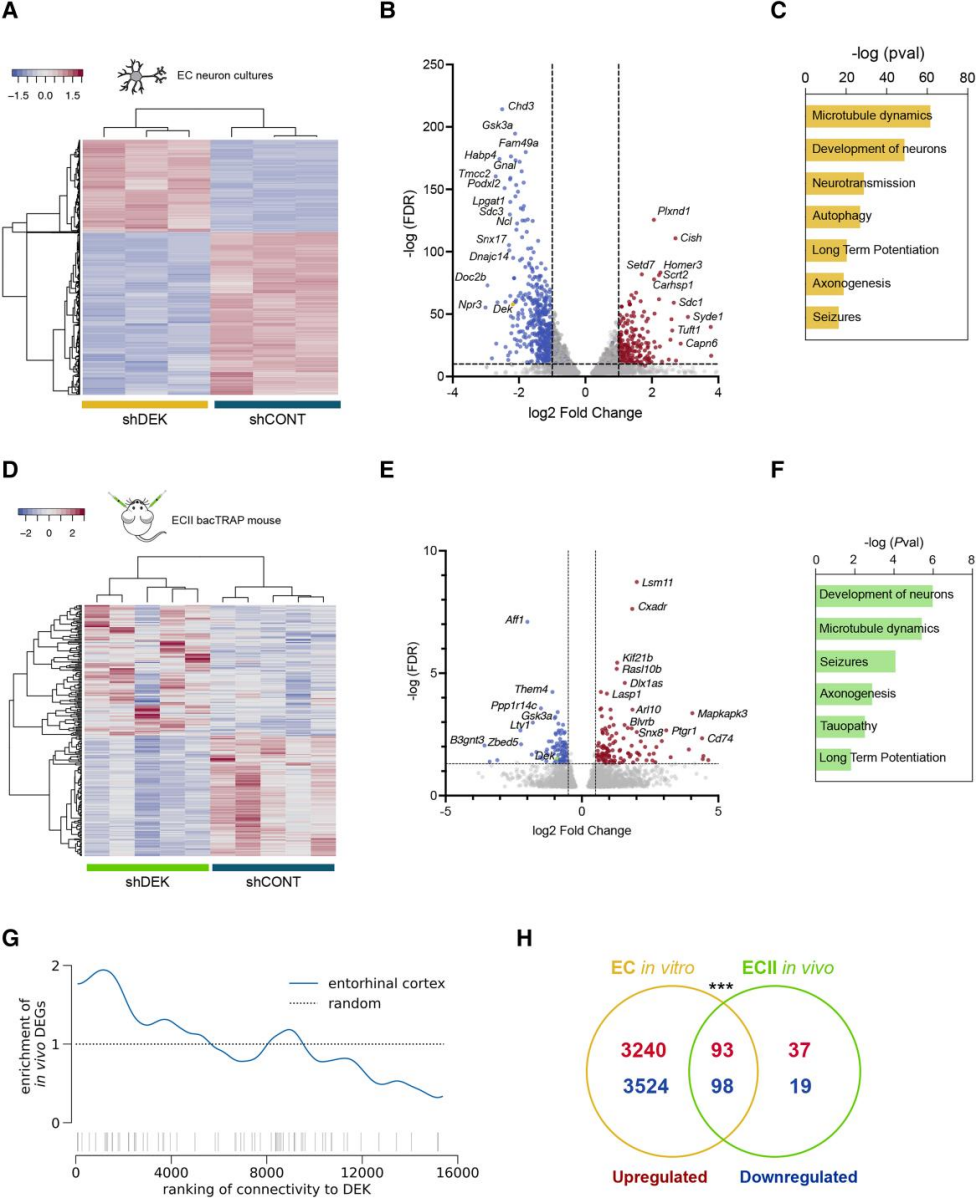
**Gene expression changes in EC neurons triggered by downregulation of *Dek in vitro* and *in vivo***. (**A**) Heat map of the differentially expressed genes (DEGs) between control (CONT) and *Dek*-silenced primary neurons (*n* = 3 wells for each group). (**B**) Volcano plot of DEGs between control and *Dek*-silenced primary neurons. (**C**) Pathways that are altered in *Dek*-silenced primary neurons compared to control. (**D**) Heat map of the DEGs between control and *Dek*-silenced ECII neurons *in vivo* (*n* = 5 mice per group). (**E**) Volcano plot of DEGs between control and *Dek*-silenced ECII neurons *in vivo*. (**F**) Pathways that are altered in *Dek*-silenced ECII neurons *in vivo* compared to control. (**G**) Enrichment analysis of DEGs [false discovery rate (FDR) < 0.05, denoted in the rug plot, *bottom*] between control and *Dek*-silenced ECII neurons *in vivo* demonstrates strong enrichment within genes ranked by probability of functional interaction with *DEK* in the EC network (blue). Dashed line represents expected enrichment if given random predictions. (**H**) Venn diagram that shows a significant overlap of up- and downregulated DEGs between *Dek*-silenced neurons *in vitro* and *in vivo*. Fisher’s exact test *P*-value < 0.001. EC = entorhinal cortex; sh = small hairpin.

**Figure 2:**
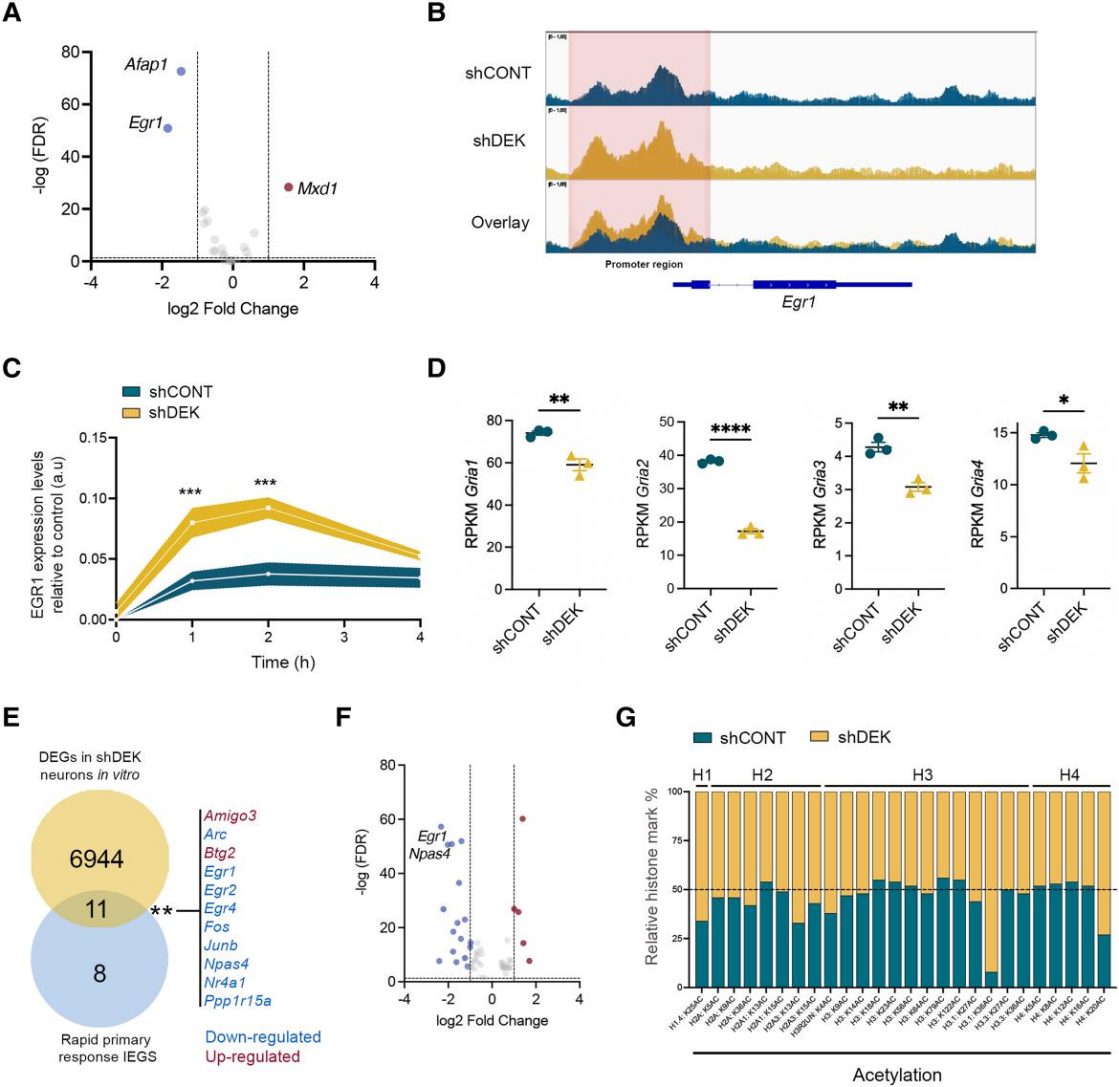
**Effect of *Dek* silencing on chromatin accessibility, immediate early gene expression and histone marks**. (**A**) Volcano plot of the differentially expressed genes (DEGs) *in vitro* that also showed alterations in chromatin accessibility at their loci (*n* = 3 wells per group). (**B**) Representative ATACseq tracks of *Dek*-silenced (yellow), control (CONT) neurons (blue) and their overlay. (**C**) Expression levels of *Egr1* after 1, 2, 3 and 4 h of induction with 50 mM KCl in control and *Dek*-silenced neurons. Two-way ANOVA Sidak’s multiple comparisons test *P*-value = 0.0006 (1 h), 0.0001 (2 h) (two independent experiments with three replicates per time point and group; additional experiments with consistent results were performed). (**D**) AMPA receptor subunits expression levels in control and *Dek*-silenced neurons *in vitro*. *P*-value = 0.0070 (*Gria1*), *P* < 0.0001 (*Gria2*), *P* = 0.0036 (*Gria3*), *P* = 0.0446 (*Gria4*). (**E**) Venn diagram of the overlap between rapid primary response immediate early genes (IEGs) and DEGs in *Dek*-silenced neurons *in vitro*. Fisher’s exact test *P*-value = 0.0095. (**F**) Volcano plot of all the IEGs that are differentially expressed in *Dek*-silenced neurons compared to controls. (**G**) Relative abundance of acetylated histone marks in control and *Dek*-silenced neurons (*n* = 1 well per group). FDR = false discovery rate; RPKM = reads per kilobase of transcript per million mapped reads.

We modulated *Dek* expression in mouse EC neuron primary cultures by transducing them with AAVs carrying either a cDNA coding for *Dek*, a silencing small hairpin RNA (shRNA) directed against *Dek*, or their respective controls. Transduction with these viruses led to a very significant overexpression and silencing of *Dek*, respectively [overexpression log2FC = 1.79, false discovery rate (FDR) = 4.33 × 10^−80^; silencing log2FC = −2.18, FDR = 1.55 × 10^−58^) (Fig. 1B, Supplementary Fig. 2C and Supplementary Table 2). This was accompanied by a significant downregulation of DEK protein levels in *Dek*-silenced neurons *in vitro* (Supplementary Fig. 4B). RNAseq followed by differential gene expression (DGE) analysis 4 days after transduction showed that *Dek* silencing has larger effects on the transcriptional landscape of the cells than DEK overexpression (Fig. 1A and B andSupplementary Fig. 2Band 2C). To study DEK function *in vivo*, we silenced *Dek* in ECII neurons of ECII-bacTRAP (bacterial artificial chromosome–translating ribosome affinity purification) mice previously generated in our laboratory (Supplementary Fig. 2A). ECII-bacTRAP mice permit immunoprecipitation and RNAseq profiling of actively translated mRNA from these neurons as previously described.^14,17^ Perturbing *Dek* expression in the EC of these mice thus allows us to measure ECII-specific gene expression regulation by DEK. BacTRAP-RNAseq analysis 1 week after AAV injection confirmed the successful silencing of *Dek* in ECII neurons (log2FC = −0.78, FDR = 0.039) (Fig. 1E and Supplementary Table 2).

We used these gene expression data to test the accuracy of the *in silico* ECII functional network. Genes predicted to be highly functionally connected to *DEK* in ECII neurons were indeed more affected by *Dek* silencing in primary EC neurons *in vitro* [*P*-value < 2.2 × 10^−16^, one-sided Wilcoxon rank sum test of DEGs FDR < 0.05 (Supplementary Fig. 3) and ECII neurons *in vivo* (*P*-value = 2.89 × 10^−11^), one-sided Wilcoxon rank sum test of DEGs FDR < 0.05, Fig. 1G] than expected by chance. These results confirm the predictive power of our functional network to reveal novel cell-type specific hub genes. We next tested whether DEK indeed regulates genes from the AD vulnerability module identified by NetWAS 2.0.^14^ Genes in the vulnerability module were indeed more differentially expressed after *Dek* silencing *in vivo* than expected by chance (*P*-value = 1.76 × 10^−07^, Fisher’s exact test), thereby validating DEK as a regulator of this module, with the potential to affect pathological mechanisms in ECII.

Pathway analysis (Ingenuity pathway analysis, DEGs FDR < 0.05) revealed that some of the most significantly downregulated pathways in *Dek*-silenced primary neurons are associated with microtubule dynamics (*P*-value = 2.94 × 10^−62^), development of neurons (*P*-value = 1.41 × 10^−49^), autophagy (*P*-value = 1.21 × 10^−27^) and axonogenesis (*P*-value = 1.50 × 10^−19^). Synaptic long-term potentiation (LTP) was also significantly decreased (*P*-value = 5.09 × 10^−21^) (Fig. 1C). Considering the modest number of significantly modified genes upon *Dek* overexpression by adjusted *P*-value, we performed pathway analysis on genes with nominally significant *P*-values (*P*-value < 0.05). These results showed that similar pathways, including LTP (*P*-value = 7.61 × 10^−5^), microtubule dynamics (*P*-value = 2.71 × 10^−11^) and development of neurons (*P*-value = 3.07 × 10^−8^) were consistently altered in *Dek*-overexpressing neurons (Supplementary Fig. 2D), supporting the notion that DEK plays a role as a modulator of synaptic transmission and plasticity in EC neurons. In agreement with the *in vitro* data, pathway analysis (Ingenuity pathway analysis, DEGs FDR < 0.05) revealed alterations in cellular functions related to microtubule dynamics (*P*-value = 3.80 × 10^−6^), development of neurons (*P*-value = 1.06 × 10^−6^) and LTP (*P*-value = 0.038) in *Dek*-silenced ECII neurons *in vivo* compared to the control (Fig. 1F). Indeed, we found a significant overlap between the DEGs in *Dek*-silenced neurons *in vitro* and *in vivo* (Fig. 1H).

### DEK epigenetically regulates the induction of *Egr1* expression

DEK has been previously identified as a chromatin-binding protein that plays a variety of roles in the regulation of chromatin structure and gene expression.^26,27^ To explore whether the effects observed on neuronal function are initiated by alterations in chromatin accessibility, we performed ATAC-seq (assay for transposase-accessible chromatin using sequencing) in primary cultures of EC neurons 4 days after *Dek* silencing. Our results showed differences in chromatin accessibility at the promoter regions of a total of 20 genes (Supplementary Table 2). These alterations were accompanied by differential expression of 13 of these genes, determined by our previous *in vitro* RNAseq analysis (Fig. 2A). Interestingly, as shown in Fig. 2A, the IEG *Egr1* was downregulated (FDR = 4.12 × 10^−48^), with the largest fold change (log2FC = −1.85) among these genes. Furthermore, as shown in our bacTRAP-RNAseq data (Supplementary Table 2), *Egr1* was the only gene that was also differentially expressed (downregulated, log2FC = −0.81) in *Dek*-silenced ECII neurons 1-week post-transduction *in vivo* (FDR = 0.013). Counterintuitively, the downregulation of *Egr1* was accompanied by increased chromatin accessibility at its proximal enhancer and promoter regions (FDR = 0.0409) (Fig. 2B).

IEG expression is induced within minutes after neuronal activation and they are essential cell-autonomous modulators of long-term neuronal plasticity and learning.^28-30^. To test the effect of DEK on Egr*1* expression dynamics, we used 50 mM KCl to cause neuronal depolarization and calcium influx in a manner that is independent of synaptic transmission. We then monitored the expression levels of *Egr1* over time using quantitative PCR. As expected, KCl caused a large transient increase in *Egr1* expression in control neurons. Strikingly though, KCl led to a 2.5-fold higher induction of *Egr1* expression in EC neurons where *Dek* was silenced compared to control (Fig. 2C). This effect is likely mediated by the higher chromatin accessibility at the proximal enhancer and promoter regions of the gene and suggests that the downregulation of *Egr1* at baseline is due to secondary alterations in neuronal activity. Indeed, secondary transcriptional changes follow to maintain homeostasis in the network since we observed decreased expression of all AMPA receptors: *Gria2* (FDR = 1.68 × 10^−52^, log2FC = −1.22), *Gria3* (FDR = 6.91 × 10^−9^, log2FC = −0.57), *Gria1* (FDR = 4.29 × 10^−7^, log2FC = −0.43) and *Gria4* (FDR = 1.23 × 10^−4^, log2FC = −0.35) (Fig. 2D), likely curbing intrinsic neuronal activity. As a consequence, we observed a significant overlap between genes differentially expressed after *Dek* silencing and a published set of rapid primary response IEGs,^31^ most of which were downregulated in *Dek*-silenced neurons (Fig. 2E). Out of all these IEGs, *Egr1* was the top downregulated gene (FDR = 4.12 × 10^−48^) (Fig. 2F).

Epigenetic modifications at the *cis* regulatory elements of IEGs are essential to regulate their expression.^32,33^ A number of studies showed a role for DEK as a histone chaperone and modulator of histone modifications, especially by regulating the function of several histone acetyltransferases and deacetylases.^34,35^ To determine specific histone marks that could be modulated by DEK in an unbiased manner, we conducted a mass spectrometry-based screen of histone post-translational modifications in control and *Dek*-silenced EC neurons *in vitro* (Supplementary Table 2). The assay revealed a striking >10-fold increase in H3.1K36ac in *Dek*-silenced neurons compared to control (Fig. 2G). While acetylation of lysine36 on this histone variant has not been extensively studied due to lack of specific antibodies, it has been reported to be located at actively transcribed genomic regions with increased chromatin accessibility on histone H3, including at the *Egr1* locus.^36-38^ To test if the increased chromatin accessibility at the *Egr1* locus after *Dek* silencing could be mediated by H3.1K36ac, we performed a CUT&Tag assay using an antibody against H3K36ac. We found no differences in H3K36ac deposition around the *Egr1* locus (Supplementary Table 2) in *Dek*-silenced neurons compared to control. As mentioned before, currently available antibodies cannot discriminate between H3.1K36ac and the more abundant H3.3K36ac (six times higher levels than H3.1K36ac in our neuron cultures and not changed by *Dek* silencing), which likely masks H3.1K36ac-specific changes. It is therefore still possible that this histone mark is responsible for the effect on *Egr1* expression. Moreover, a reader protein of H3K36ac, Setd7^39^ is very significantly induced both *in vitro* and *in vivo* after *Dek* silencing (log2FC = 1.69, *P_adj_* = 1.2 × 10^−82^; log2FC = 0.58, *P_adj_* = 0.012, respectively), suggesting an important role of this histone mark in mediating DEK function.

### *Dek*-silenced entorhinal cortex neurons show altered intrinsic excitability

**Figure 3:**
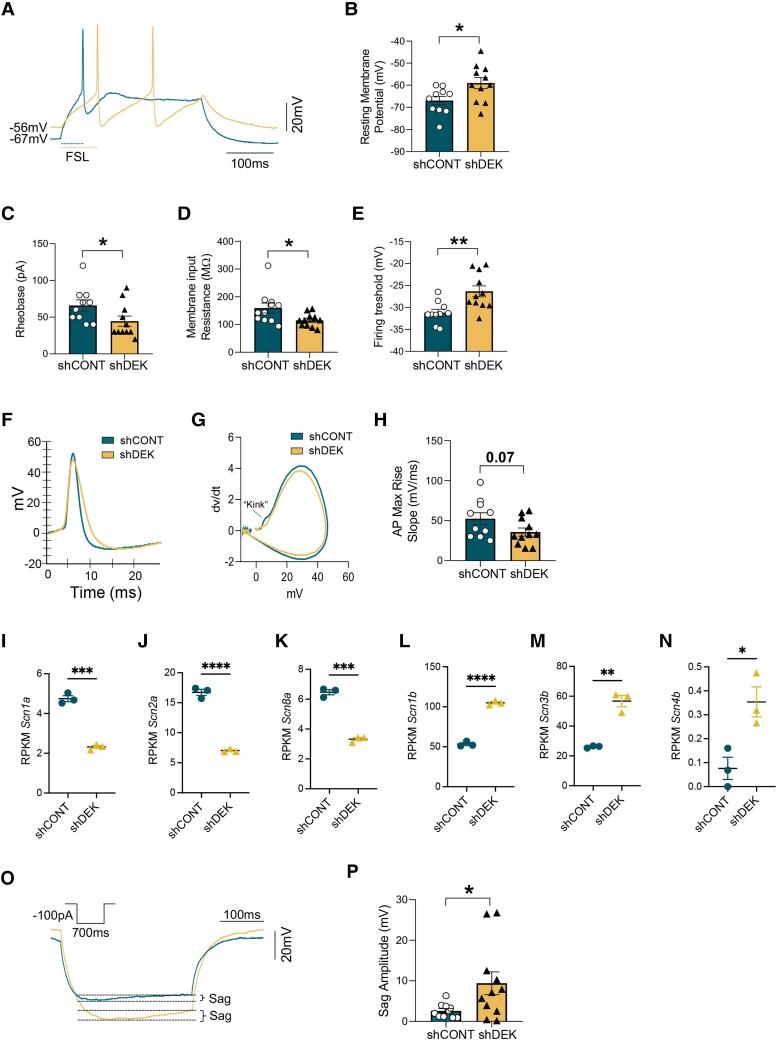
**Electrophysiological properties of *Dek*-silenced EC primary neurons**. (**A**) Representative traces of first spike and resting membrane potential from: control (CONT, blue) and *Dek*-silenced neurons (yellow) (*n* = 10–11 neurons per group). (**B**) Bar graph of the resting membrane potential in control versus *Dek*-silenced neurons. Unpaired *t*-test *P*-value = 0.023. (**C**) Bar graph of the rheobase in control versus *Dek*-silenced neurons. Mann-Whitney test *P*-value = 0.037. (**D**) Bar graph of the membrane input resistance in control versus *Dek*-silenced neurons. Mann-Whitney test *P*-value = 0.034. (**E**) Bar graph of the firing threshold in control versus *Dek*-silenced neurons. Unpaired *t*-test *P*-value = 0.0046. (**F**) Representative action potential (AP) waveform in control and *Dek*-silenced neurons. (**G**) Representative phase plot in control and *Dek*-silenced neurons. (**H**) Bar graph of the AP maximum rise slope in control versus *Dek*-silenced neurons. Unpaired *t*-test *P*-value = 0.07. (**I–N**) Expression levels of the differentially expressed potassium and calcium channel subunits between control and *Dek*-silenced entorhinal cortex (EC) primary neurons (*n* = 3 wells for each group). Unpaired *t*-test *P*-value = 0.0001 (*Scn1a*), *P* < 0.0001 (*Scn2a*), *P* = 0.0001 (*Scn8a*), *P* < 0.0001 (*Scn1b*), *P* = 0.0013 (*Scn3b*), *P* = 0.024 (*Scn4b*). (**O**) Representative traces of 100 pA hyperpolarizing current from control and *Dek*-silenced neurons. (**P**) Bar graph of Sag amplitude in control and *Dek*-silenced neurons. Unpaired *t*-test *P*-value = 0.050. RPKM = reads per kilobase of transcript per million mapped reads.

The modulation of IEGs by DEK could have profound consequences for neuronal function. To test this hypothesis, we performed patch-clamp recordings in *Dek*-silenced EC neurons in primary culture 4 days after transduction. Evaluation of the RMP showed that *Dek*-silenced neurons were significantly hypopolarized compared to controls (Fig. 3A and B). Concurrently, the rheobase (minimal current required to elicit an action potential), was lower, suggesting increased excitability (Fig. 3C). Decreased membrane input resistance and increased firing threshold (Fig. 3D and E) confirmed alterations in the neuronal intrinsic properties (Supplementary Table 3). These results suggest a functional change in Na^+^ currents. The conductance of voltage-gated sodium channels can be further evaluated by determining the kinetics of the AP initiation.^40,41^ We did not find alterations in the AP waveform (Fig. 3F). However, in agreement with alterations in voltage-gated sodium channels, we observed a lack of the first spike initiation, or ‘kink’ (Fig. 3G) and a trend for a decrease in the AP MRS (Fig. 3H). Likely underlying these changes, the expression of voltage-gated sodium and potassium channel subunits was broadly dysregulated (Fig. 3I–N and Supplementary Fig. 4A).

Last, we found a significant increase in the sag amplitude (Fig. 3O and P), the difference between initial and steady state decrease in voltage after a hyperpolarizing current step, a key contributor to the RMP.^42,43^ Taken together, these data strongly indicate that DEK can cell-autonomously regulate ECII neuron excitability. Interestingly, increased sag amplitude, together with a hypopolarized membrane potential, were previously found in neurons with pathological tau accumulation.^44-46^ Our ECII functional network also predicted a strong connection between DEK and tau. We therefore hypothesized that DEK might be a molecular bridge between synaptic homeostasis and tau proteostasis, and that, like at early stages of AD, *Dek*-silenced neurons might have elevated tau in addition to electrophysiological abnormalities.

### *Dek* silencing leads to tau accumulation, microglia reactivity and ECII neuron loss

**Figure 4:**
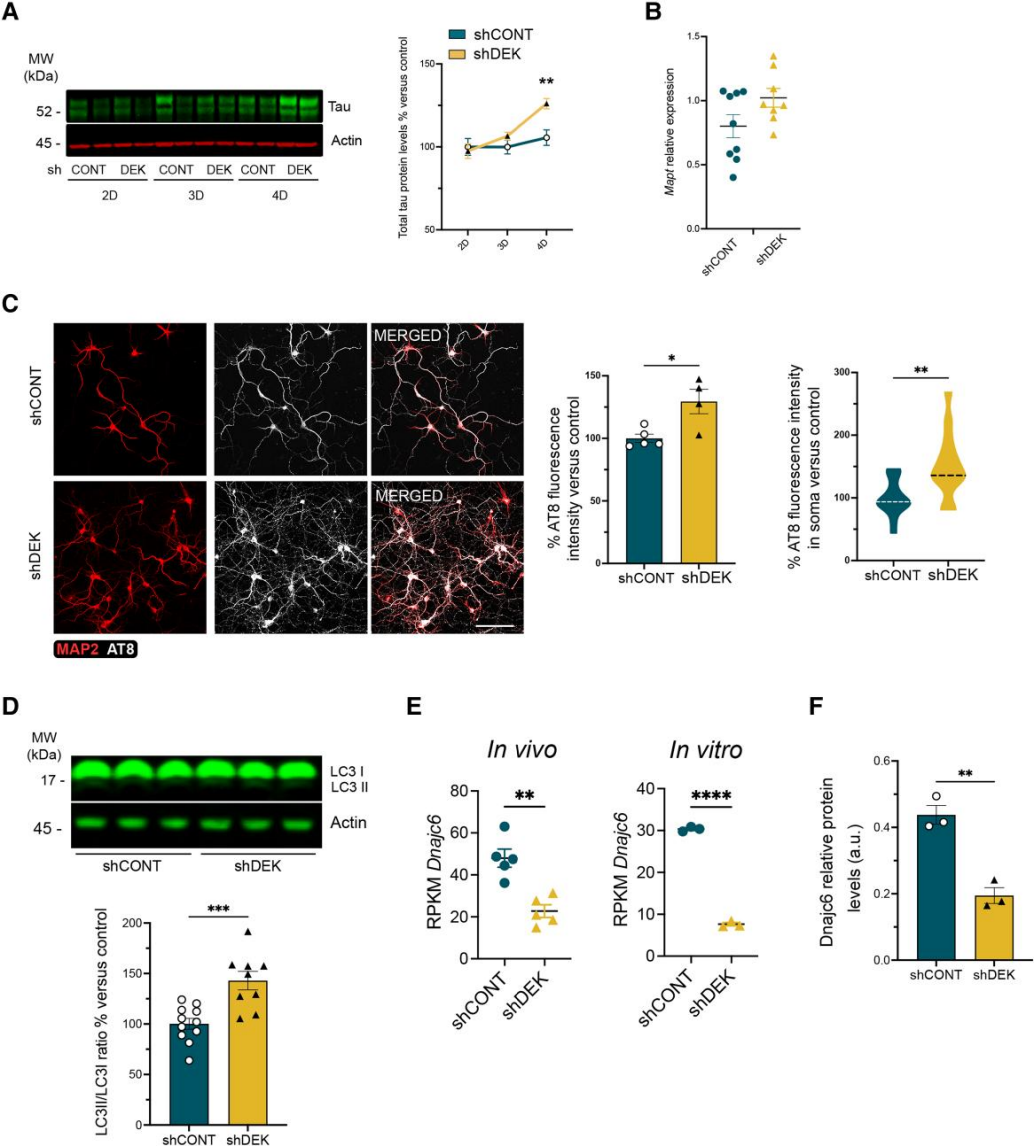
**Effect of *Dek* silencing in EC neurons *in vitro***. (**A**) Western blot analysis of the levels of total tau (green) in entorhinal cortex (EC) neurons in primary culture at 2, 3 and 4 days post-transduction (one representative experiment is shown with *n* = 2 replicates per time point per group). The graphs show protein levels in % relative to 2 days post-transduction control neurons. Band intensity was normalized with actin for total tau measurements and with total tau for phospho-tau measurements. Two-way ANOVA Sidak’s multiple comparisons test *P*-value = 0.0019 (three independent experiments with two replicates per time point and group). (**B**) RT-qPCR analysis of *Mapt* expression levels in control and *Dek*-silenced neurons 4 days after transduction (three independent experiments with three replicates per group). (**C**) Confocal microscopy images of immunofluorescence staining of MAP2 (red) and AT8 (greyscale) in control (CONT) and *Dek*-silenced neurons at 4 days post-transduction. Scale bar = 100 μm. The graphs show AT8 fluorescence intensity levels in % relative to control-treated neurons. Unpaired *t*-test *P*-value = 0.017 (total AT8), *P* = 0.0055 (somatic AT8) (AT8 total fluorescence graph shows the relative fluorescence intensity per field from one experiment and 4–5 replicates per condition. Additional experiments with consistent results were performed. Distribution of AT8 fluorescence in the soma was determined in two independent experiments with 4–5 replicates each, *n* = 40–60 neurons per group in total). (**D**) Western blot analysis of LC3 (three independent experiments and 3–4 replicates per experiment and group). *P*-value = 0.0006. *Dnajc6* expression levels in control and *Dek*-silenced neurons *in vivo* and *in vitro*. Unpaired *t*-test *P*-value = 0.0014 (*in vivo*), *P* < 0.0001 (*in vitro*). (**F**) Dnajc6/auxilin relative protein levels in control and *Dek*-silenced neurons *in vitro* (*n* = 3 wells per group). Unpaired *t*-test *P*-value = 0.0028. LC = locus coeruleus; MW = molecular weight; RPKM = reads per kilobase of transcript per million mapped reads; sh = small hairpin.

To assess tau accumulation, we measured total tau protein levels and its phosphorylation by western blot at different time points following *Dek* silencing in EC neurons in primary culture. As shown in Fig. 4A, *Dek* silencing led to increased tau levels 4 days after transduction, accompanied by its redistribution to the somatodendritic compartment (Fig. 4C), an early event on AD progression.^47,48^ These alterations were not accompanied by increased tau phosphorylation at Thr231, generally associated with early AD pathology, or at Ser202/Thr205, associated with later stages of AD pathology^49,50^ (Supplementary Fig. 4C ad D). But a significant decrease in phosphorylation at another early AD epitope, Thr181 (Supplementary Fig. 4E) suggested a subtle imbalance in the phosphorylation profile of tau. RT-qPCR analysis showed no differences in tau mRNA levels (Fig. 4B), suggesting that *Dek* silencing triggers tau accumulation by modulating its translation, release or degradation. Pathway analysis on our *in vitro* RNAseq data showed autophagy alterations in *Dek*-silenced primary EC neurons (Fig. 1C). To test if *Dek* silencing indeed affects autophagy in these neurons, we performed western blot analysis of the protein LC3, the lipidation of which initiates autophagosome formation. *Dek*-silenced neurons showed increased levels of the lipidated form LC3-II, which supports the notion of an altered autophagic flux (Fig. 4D). Interestingly, *Dnajc6*, which codes for auxilin, a protein necessary for lysosomal degradation of autophagosome content with mutations associated with Parkinson’s disease,^51^ was among the most significantly downregulated genes in *Dek*-silenced EC neurons both *in vitro* and *in vivo* (log2FC = −2.1, *P_adj_* = 4.9 × 10^−145^; log2FC = −0.6, *P_adj_* = 0.008, respectively). Mass spectrometry proteomics analysis of *Dek*-silenced neurons *in vitro* further showed a significant decrease of auxilin protein abundance in these cells (log2FC = − 2.25, *P*-value = 0.003), confirming its regulation by DEK (Supplementary Table 2).

**Figure 5:**
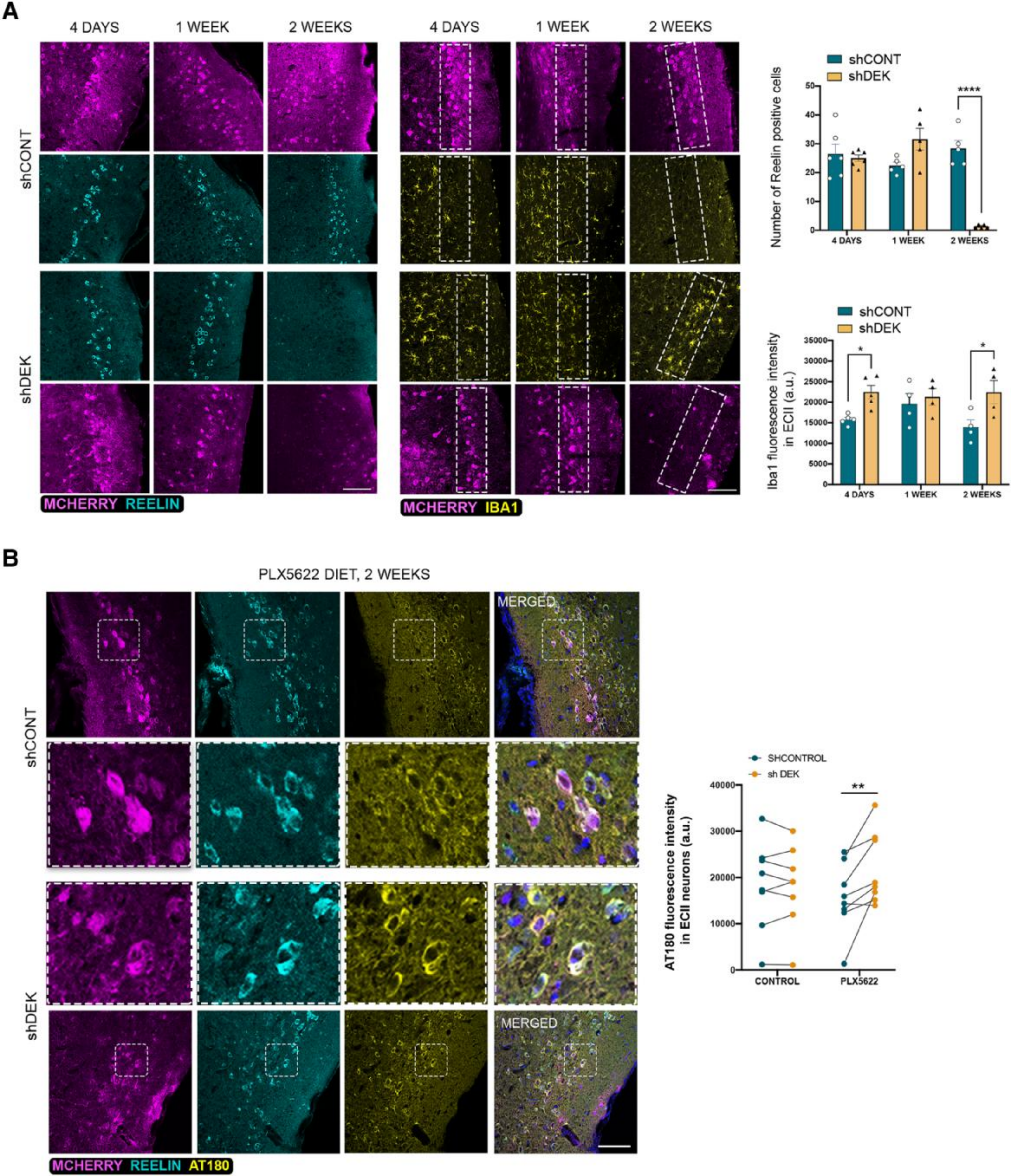
**Effect of *Dek* silencing in ECII neurons of hMAPT mouse**. (**A**) Confocal microscopy images of immunofluorescence staining of transduced neurons (mCherry, magenta), ECII neurons (Reelin, cyan) and microglia (Iba1, yellow) at 4 days, 1 week and 2 weeks post-transduction of human-tau transgenic (hMAPT) mice. Scale bar = 100 μm. The graphs show the quantifications of the number of reelin positive cells and of Iba1 fluorescence intensity in layer II of the mouse entorhinal cortex (EC) in the control (CONT) and *Dek*-silenced hemispheres. Two-way ANOVA Sidak’s multiple comparisons test *P*-value < 0.0001 (Reelin, 2 weeks), *P* = 0.044 (Iba1, 4 days), *P* = 0.021 (Iba1, 2 weeks) (*n* = 4–6 sections per time point per group from two different animals). (**B**) Confocal microscopy images of immunofluorescence staining of transduced neurons (mCherry, magenta), ECII neurons (Reelin, cyan) and phospho-tau Thr231 (AT180, yellow) at 2 weeks post-transduction of control or sh*Dek*-carrying AAVs in PLX5622 diet fed hMAPT mice. Scale bar = 100 μm. The graph shows the quantification of AT180 fluorescence intensity, paired by the control and the *Dek*-silenced opposite hemisphere for each hMAPT mouse. Two-way ANOVA Sidak’s multiple comparisons test *P*-value = 0.0014 (*n* = 8 mice per group). sh = small hairpin.

To test whether these alterations also lead to tau pathology in ECII neurons *in vivo*, we performed stereotaxic injections of a control and a *Dek* silencing AAV in ECs in hMAPT mice. This mouse model is knocked-out for mouse tau while expressing the full length human MAPT gene.^16^ Immunofluorescence labelling of ECII neurons using the Reelin marker at 4 days, 1 week and 2 weeks after injection (Supplementary Fig. 5A**)** revealed a highly significant loss of these neurons after 2 weeks (Fig. 5A). Iba1 immunofluorescence staining showed increased reactive microglia specifically in the layer II of the EC at 2 weeks post-injection (Fig. 5A). At 1-week post-injection (before neuron loss), we found no differences in tau protein levels in ECII by immunofluorescence against phospho-tau (Thr231) (Supplementary Fig. 5B). Accumulation of reactive microglia around transduced neuron cell bodies (Supplementary Fig. 5C) suggested that microglia might contribute to the neuron loss observed at 2 weeks post-injection. Indeed, RT-qPCR on EC lysates showed increased expression of the microglia markers *Cx3cr1*, *Itgam* (that codes for CD11b) and complement C*1qa* (associated with synaptic pruning)^52,53^ (Supplementary Fig. 5D–F). We also found an increase in the disease-associated microglia genes *Tyrobp* and *Spp1*^54,55^ (Supplementary Fig. 5G and H). Considering that our previous data suggest that *Dek*-silenced neurons exhibit increased excitability, we measured the levels of *Ccl3*, a cytokine shown to be upregulated in microglia in response to neuronal activation^56^ (Supplementary Fig. 5I). The significant upregulation of *Ccl3* suggested that ECII hyperexcitability contributes to microglial reactivity.

To test the possibility that a longer time is necessary for progressive build-up of tau, we eliminated microglia to increase ECII neuron survival. We fed mice with chow containing PLX5622, an antagonist of colony-stimulating factor 1 receptor (CSF1R), that leads to microglia depletion after 5 days.^57^ At Day 5, we injected control or sh*Dek* AAVs in the EC of opposite hemispheres and analysed ECII neuron survival and tau accumulation at 2 weeks post-injection (Supplementary Fig. 6A). As shown in Supplementary Fig. 6B and C, PLX5622 successfully ablated microglia and rescued the loss of *Dek*-silenced ECII neurons. We next compared the somatic levels of phospho-tau (Thr231) in layer II of the EC between control and *Dek* silenced entorhinal cortices in PLX5622-treated mice and found a significant accumulation of tau following *Dek* silencing (Fig. 5B).

### Dysregulation of *DEK* in human Alzheimer’s disease

To test if *DEK* is dysregulated at the transcriptional level in early AD in EC neurons, we consulted a recently published single-nucleus RNAseq dataset, but this study did not detect differential expression of *DEK*. We then mined the NHGRI-EBI GWAS catalogue for genetic variations in *DEK* that could be associated with AD. Interestingly, an SNP in a *DEK* intron, rs145578678, was recently found to have a suggestive association with the rate of cognitive decline in AD (*P*-value = 3 × 10^−6^).^58^ To assess the functional effect that this putative disease-associated allele may have, we used DeepSEA, a deep learning-based algorithm that predicts the chromatin effects of sequence alterations,^59^ using rs145578678 and SNPs in LD with it as input. After filtering for observations specific to brain, H3K36me3 was predicted to be increased in the hippocampus for the disease-associated allele of rs182353546, within an intron of *DEK* (Supplementary Table 2). H3K36me3 and H3K36ac are mutually exclusive.^36,60^ As H3K36ac is the histone modification most affected by DEK, it is likely that, in a feedback loop, DEK regulates H3K36ac deposition in its own loci to regulate its transcriptional level. The *DEK* allele putatively associated with cognitive decline in AD might therefore improperly regulate DEK levels and perturb proper synaptic homeostasis and tau regulation.

## Discussion

The molecular cascade by which ADdrivers and genetic susceptibility factors result in early tau pathology in specific neuronal subpopulations remains to be understood. In the present work, we set out to identify these molecular processes by using a cell type-specific functional genomics approach. A feature of this framework, compared to previous studies,^61-63^ is that it models gene networks for a unique neuron type as opposed to a whole brain region, and it is the first one to integrate quantitative genetics^11^ and *in vivo* genomics data for AD vulnerable neurons.^61-63^ It is, thus likely to identify genes that are indeed regulating tau during the natural course of human AD within ECII neurons, especially during early disease stages.

By using this approach, we take a significant step towards understanding the neuronal arm of AD pathogenesis: we identify perturbations in DEK as a novel molecular bridge between a wide range of processes that take place during early stages of AD: dysregulation of neuronal excitability, tau accumulation and alterations in microglia activity (Supplementary Fig. 7). While we did not find altered *DEK* expression in neurons in a recent single cell transcriptomics study of the EC in human AD,^54^ its function can be regulated by post-translational modifications^64^ and by degradation.^65^ Further studies will be necessary to assess DEK activity levels during prodromal AD, as well as to elucidate the functional impact of genetic variation in DEK, like the rs145578678 polymorphism. It will also be important to validate the cell type specificity of the pathological cascade mediated by ubiquitously-expressed DEK and determine if DEK also regulates tau accumulation in LC or in hippocampal neurons. Whether the perturbation of DEK activity by prodromal AD or its downstream consequence occur in a cell type-specific manner is an open question.

Here we perform an in-depth characterization of DEK function in vulnerable neurons and report that its silencing potentiates activity-regulated gene expression. At steady state, this results in decreased IEG expression, likely due to homeostatic changes aimed to normalize what is sensed as aberrant network activity following chronic upregulation of IEGs. The existence of these compensatory events is supported by our data showing a downregulation of all AMPA glutamate receptors subunits that would, in turn, decrease IEG expression. Together with the perturbation of genes involved in microtubule dynamics, this suggests that DEK regulates synaptic homeostasis in ECII neurons, a central process in their associative memory-forming function.

Our results further show that the alterations in neuronal activity mediated by *Dek* silencing trigger neuron-to-microglia signalling mechanisms that steer microglia towards a pro-inflammatory and phagocytic state that contributes to neurodegeneration, as demonstrated by the rescue of neuron loss by microglia depletion. Microglia reactivity preceded accumulation of pThr231-tau *in vivo*. Although we cannot exclude that neuronal accumulation of another tau proteoform is responsible for altering microglial state, our data indicate that it might be due, instead, to the detection of other neuronal dysfunction, like excessive firing. This may represent an extreme case of the spine phagocytosis described after neuronal hyperactivation.^66^ In agreement with this notion, we found elevated levels of *Ccl3*, a cytokine upregulated by microglia in response to neuronal activity^56^ and of known mediators of synaptic spine loss (*Itgam*, *C1qa*).^52,53,67,68^ While we also found increased expression of *Tyrobp* and *Spp1*, both upregulated in disease-associated microglia in AD,^54,55,69^ a more comprehensive analysis of gene expression changes at single cell resolution will be necessary to determine which microglia states are triggered by *Dek* silencing in ECII neurons. This neuron-glia crosstalk regulated by DEK in neurons might take centre stage during early AD pathology in the EC. Follow-up studies to identify the neuronal signal regulated by DEK that drives the elimination of hyperexcitable ECII neurons, will be of great relevance, as these signalling molecules may serve as potential therapeutic targets for early intervention in AD.

Many AD drivers, such as ageing, amyloid and genetic risk factors like APOE E4, perturb synaptic homeostasis and increase neuronal excitability.^70-76^ Indeed, epileptiform activity in the hippocampus is a very early feature of AD.^77-79^ In turn, hyperactivity of the hippocampal formation exacerbates pathological lesions.^80-85^ However, the connection between AD lesions and the disruption of synaptic homeostasis in the entorhinal-hippocampal network has remained elusive. It is possible that the regulation of tau levels is an integral part of a physiological programme of structural and synaptic plasticity. Indeed, a recent transcriptional analysis of NFT-bearing neurons in human AD at single-cell resolution suggested that the build-up of NFT is a cell type-specific response to alterations in synaptic function.^86^ Improper fine-tuning of these crucial mechanisms for ECII function might contribute to the onset of a deadly cascade in ECII neurons. This notion is supported by the progressive tau build-up that we observe in *Dek*-silenced neurons both *in vitro* and *in vivo*. *In vitro*, tau accumulation and proteoform imbalance is accompanied by an increase in LC3 lipidation suggestive of alterations in the autophagy flux, and a downregulation of *Dnajc6*/auxilin both at the transcriptional and protein levels, which might cause an impairment in autophagosomal content degradation. While these data point to autophagy alterations as contributing factor to the build-up of tau in *Dek*-silenced neurons, other mechanisms could further participate in this process. For instance, neuronal activity has been shown to affect tau translation, degradation and secretion,^83,85,87,88^ suggesting that the electrophysiological phenotype caused by impaired DEK signalling might contribute to tau accumulation through different mechanisms that will need to be explored in future studies. A large body of literature has shown that amyloid accumulation in the EC affects ECII excitability.^73,89-92^ According to our data, if ECII neurons adjust DEK activity to re-establish proper excitability, this might consequently lead to impairments in tau proteostasis. Understanding how this novel axis is affected by amyloid accumulation and ageing will be crucial to dissect the molecular steps that take place specifically in these neurons during prodromal AD.

## Supplementary Material

awae051_Supplementary_Data

## Data Availability

The sequencing data generated in this article are available on Gene Expression Omnibus with the superseries accession number GSE203368. Accession numbers for CUT&Tag/ATACseq, *in vivo* RNAseq and *in vitro* RNAseq data are GSE203364, GSE203365 and GSE203366, respectively.
