# Supplementary material for: A cell autonomous regulator of neuronal excitability modulates tau in Alzheimer’s disease vulnerable neurons
Source: Brain. 2024 Mar 11;147(7):2384–99. doi: 10.1093/brain/awae051 (PMC11224620; doi:10.1093/brain/awae051)
Supplement: awae051_Supplementary_Data [file awae051_supplementary_data.zip › brain-2023-02150-File007.pdf]

# Supplementary materials and methods

## ATAC-seq

ATAC-seq was performed as previously described [1]. Briefly 50,000 EC primary neurons were pelleted by centrifugation (5 min, 500 xg at 4°C) and lysed in 10 mM Tris-HCl pH 7.4, 10 mM NaCl, 3 mM $MgCl_2$, 0.1% NP-40, 0.1% Tween-20 and 0.01% digitonin. Nuclei where then pelleted by centrifugation (10 min, 1000 xg at 4°C) and resuspended in transposition reaction mix: 1% digitonin, 10% Tween-20, Tn5 transposase and TD buffer (Nextera DNA Library Prep Kit, #FC-121-1030, Illumina). After transposase reaction (30 min, 37°C, 1000 RPM) and DNA purification (MinElute Reaction Cleanup Kit, #28204, Qiagen), the transposed samples were amplified by PCR with individual barcode primers per sample (NEB Next High-Fidelity 2x PCR Master Mix, #M0541S, NEB). After 5 PCR cycles, a fraction of the partially amplified library was quantified by qPCR to calculate the number of additional PCR cycles needed to amplify each sample without saturation (number of cycles needed to reach 1/3 of the maximum R). The amplified libraries were purified with Agencourt AMPure XP magnetic beads (#A63880, Beckman Coulter) and their quality and concentration were determined with an Agilent High Sensitivity DNA bioanalysis Chip. Libraries were sequenced on a NextSeq sequencer (Illumina).

## Mass spectrometry analysis of histone modifications

Mass spectrometry analysis of histone posttranslational modification (Mod-spec) was performed by Activ Motif on primary EC neurons transduced with control or Dek-silencing AAVs. Bulk histones were acid-extracted from pellets obtained from EC neurons in primary culture transduced with either control or *Dek*-silencing AAVs, propionylated and subjected to trypsin digestion as described previously [2]. Briefly, histones were extracted at room temperature for 1 h in 0.2M sulfuric acid with intermittent vortexing. Histones were then precipitated by the addition of trichloroacetic acid (TCA) on ice, and recovered by centrifugation (10,000 x g, 5 min at 4°C). The pellet was then washed once with 1 mL cold acetone/0.1% HCl, twice with 100% acetone, and air dried. Histones were propionylated with 1:3 v/v propionic anhydride/2-propanol and incremental addition of ammonium hydroxide to keep the pH around 8, and subsequently dried in a SpeedVac concentrator. The pellet was then resuspended in 100 mM ammonium bicarbonate and adjusted to pH 7-8 with ammonium hydroxide. Histones were then digested with trypsin resuspended in 100 mM ammonium

bicarbonate overnight at 37°C and dried in a SpeedVac concentrator. The pellet was resuspended in 100 mM ammonium bicarbonate and propionylated a second time as described above. Histone peptides were resuspended in 0.1% TFA in $H_2O$ for mass spectrometry analysis.

Samples were analyzed on a triple quadrupole (QqQ) mass spectrometer (Thermo Fisher Scientific TSQ Quantiva) directly coupled with an UltiMate 3000 Dionex nano-liquid chromatography system. Peptides were loaded onto an in-house packed trapping column (3 cm×150 µm) and separated on a New Objectives PicoChip analytical column (10 cm×75 µm). Both columns were packed with New Objectives ProntoSIL C18-AQ, 3 µm, 200 Å resin. The chromatography gradient was achieved by increasing percentage of buffer B from 0 to 35% at a flow rate of 0.30 µl/min over 45 min. Solvent A: 0.1% formic acid in water, and B: 0.1% formic acid (FA) in 95% acetonitrile (ACN). The QqQ settings were as follows: collision gas pressure of 1.5 mTorr; Q1 peak width of 0.7 (FWHM); cycle time of 2 s; skimmer offset of 10 V; electrospray voltage of 2.5 kV. Targeted analysis of unmodified and various modified histone peptides was performed. This entire process was repeated three separate times for each sample.

### Data analysis

Raw MS files were analyzed in Skyline with Savitzky-Golay smoothing [3]. All Skyline peak area assignments for monitored peptide transitions were manually confirmed. Multiple peptide transitions were quantified for each modification. For each monitored amino acid residue, each modified (and unmodified) form was quantified by first calculating the sum of peak areas of corresponding peptide transitions; the sum of all modified forms was then calculated for each amino acid to represent the total pool of modifications for that residue. Each modification is represented as a percentage of the total pool of modifications. This process was carried out for each of the three separate mass spec runs, and the raw data corresponds to the mean and standard deviation of the resulting three values from this analysis for each modified and unmodified form of the corresponding amino acid residue.

## CUT&Tag

Frozen cell pellets from control and *Dek*-silenced EC mouse primary neurons were sent to Active Motif for CUT&Tag. Briefly, cells were washed and incubated overnight with Concanavalin A beads and 1.3 µl/reaction of primary antibody against H3K36Ac (#39379,

Activ Motif). After incubation with the secondary anti-rabbit antibody (1:100), cells were washed and tagmentation was performed at 37°C using protein-A-Tn5. Tagmentation was halted by the addition of EDTA, SDS and proteinase K after which DNA extraction and ethanol purification was performed, followed by PCR amplification and barcoding (see Active Motif CUT&Tag kit, catalog number 53160 for recommended conditions and indexes). Following SPRI bead cleanup (Beckman Coulter), the resulting DNA libraries were quantified and sequenced on Illumina's NextSeq 550 (8 million reads, 38 paired end).

## Data analysis

The paired-end 38 bp sequencing reads generated by Illumina sequencing were mapped to the genome using the BWA algorithm with default settings. Only reads that passed Illumina's purity filter, aligned with no more than 2 mismatches, and mapped uniquely to the genome were used to subsequent analysis. Duplicate reads were removed. Genomic regions with high levels of transposition/tagging events were determined using the MACS2 peak calling algorithm. To identify the density of transposition events along the genome, the genome was divided into 32 bp bins and the number of fragments in each bin was determined by extending the reads to 200 bp, which is close to the average length of the sequenced library inserts. To compare peak metrics between samples, overlapping intervals were grouped into "merged regions" which are defined by the start coordinate of the most upstream interval and the end coordinate of the most downstream interval. In locations where only one samples has an interval, that interval defined the merged region.

## bacTRAP-RNAseq

Tissue was homogenized on 1ml of lysis buffer (20 mM Hepes KOH, 10 mM $MgCl_2$, 150 mM KCl, 0.5 mM DTT, 100 µg/ml cycloheximide) supplemented with protease (#A32965, ThermoFisher) and RNAse inhibitors (40U/ml RNAsin, #N2515, Promega and 20U/ml Superasin, #AM2696, ThermoFisher) at 4°C in a glass Teflon homogenizer. After centrifugation, the supernatant was incubated with 1% NP-40 and 30 mM DHPC (#850306P, Avanti) on ice for 5 min. After centrifugation, the supernatant, containing ribosome bound RNAs, was incubated over-night with magnetic beads (Streptavidin MyOne T1 Dynabeads, #65602, ThermoFisher) previously coated with anti EGFP antibodies for immunoprecipitation (HtzGFP-19C8 and HtzGFP-19F7, from MSKCC monoclonal antibody facility [4]). Immunoprecipitated RNAs were then purified using the RNeasy Plus Micro Kit (#74034, Qiagen). RNA integrity was determined with a Bioanalyzer 2100 (Agilent) using an RNA 6000 pico chip (#5067-1513, Agilent). RNA was quantified with Quant-it Ribogreen RNA reagent

(#R11490, ThermoFisher). Reverse transcription was performed with Ovation RNAseq v2 kit (#7102, NuGEN) from 5 ng of RNA following the manufacturer's instructions. cDNAs were purified using the QIAquick PCR purification kit (#28104, Qiagen). cDNA yield was measured with Quant-IT Picogreen dsDNA kit (#P7581, ThermoFisher). 200 ng of cDNA were used for fragmentation prior to cDNA library preparation. cDNA was sonicated into 200 bp fragments using a Covaris S2 ultrasonicator instrument (10% duty cycle, intensity 5, 200 cycles/burst per second for 2 min at 5.5°C to 6°C). Library preparation was performed with the TruSeq RNA sample preparation kit v2 (#RS-122-2001, Illumina) and were sequenced at the Rockefeller University genomics resource center on a NextSeq 500 sequencer (Illumina).

## Mass spectrometry analysis of Cells

### Sample preparation

Cell pellets were thawed on ice and lysed with addition of 80 µl of 8M urea and 0.1% ProteaseMAX™ surfactant (Promega) in 100 mM Tris-HCl, pH 8.5, mixed 1 µl protease inhibitor cocktail (Roche) and sonicated in water bath for 10 min. The samples were sonicated using VibraCell probe (Sonics & Materials, Inc.) for 20 s with pulse 2/2 (on/off), at 20% amplitude. Lysates were spun down at 12,000 rpm at 4°C for 10 min and protein concentration was determined by BCA assay (Pierce) and a volume corresponding to 25 µg of protein was taken and supplemented with Tris-HCl buffer up to 99 µl and additional 11 µl ACN. Proteins were reduced by adding 1.1 µl of 500 mM dithiothreitol (Sigma) and incubated at 25°C for 60 min while shaking at 400 rpm on a block heater. Alkylation was performed with addition of 3.3 µl of 500 mM iodoacetamide (Sigma) at room temperature for 60 min at 400 rpm in dark. Then 1 µg of sequencing grade modified trypsin (Promega) was added to the samples and incubated for 16 h at 37°C.  The digestion was stopped with 22 µl cc. FA, incubating the solutions at RT for 5 min. The sample was cleaned on a C18 Hypersep plate with 40 µl bed volume (Thermo Fisher Scientific), dried using a vacuum concentrator (Eppendorf) and resuspended in 25 µl of 0.1% formic acid and 2% ACN.

### Liquid Chromatography-Tandem Mass Spectrometry Data Acquisition

Peptides were reconstituted in solvent A and approximately, two µg samples injected on a 50 cm long EASY-Spray C18 column (Thermo Fisher Scientific) connected to an Ultimate 3000 nanoUPLC system (Thermo Fisher Scientific) using a 120 min long gradient: 4-26% of

solvent B (98% ACN, 0.1% FA) in 120 min, 26-95% in 5 min, and 95% of solvent B for 5 min at a flow rate of 300 nL/min. Mass spectra were acquired Q Exactive HF hybrid quandrupole-Orbitrap mass spectrometer (Thermo Fisher Scientific) ranging from *m/z* 375 to 1500 at a resolution of R=120,000 (at *m/z* 200) targeting $1x10^6$ ions for maximum injection time of 86 ms, followed by data-dependent higher-energy collisional dissociation (HCD) fragmentations of top 17 precursor ions with a charge state 2+ to 8+, using 60 s dynamic exclusion. The tandem mass spectra were acquired with a resolution of R=30,000, targeting $5x10^5$ ions for maximum injection time of 54 ms, setting quadrupole isolation width to 1.4 Th and normalized collision energy to 28%.

## Data Analysis

Acquired raw data files were analyzed using Proteome Discoverer v2.4 (Thermo Fisher Scientific) with MS Amanda v2.0 search engine against mouse protein database (SwissProt, 55,310 entries downloaded on 1 December 2021). A maximum of two missed cleavage sites were allowed for full tryptic digestion, while setting the precursor and the fragment ion mass tolerance to 10 ppm and 0.02, respectively. Carbamidomethylation of cysteine was specified as a fixed modification. Oxidation on methionine, deamidation of asparagine and glutamine as well as acetylation of lysine and peptide N-termini were set as dynamic modifications. Initial search results were filtered with 5% FDR using Percolator node in Proteome Discoverer. Quantification was based on the precursor ion intensities.

## References


1. Buenrostro JD, Wu B, Chang HY, & Greenleaf W J. (2015). ATAC-seq: A method for assaying chromatin accessibility genome-wide. *Current Protocols in Molecular Biology*, *2015*, 21.29.1-21.29.9. https://doi.org/10.1002/0471142727.mb2129s109
2. MacLean B., Tomazela DM, Shulman N et al. (2010). Skyline: An open source document editor for creating and analyzing targeted proteomics experiments. *Bioinformatics*, *26*(7), 966–968. https://doi.org/10.1093/bioinformatics/btq054
3. Zheng Y, Thomas PM, & Kelleher NL. (2013). Measurement of acetylation turnover at distinct lysines in human histones identifies long-lived acetylation sites. *Nature Communications*, *4*, 1–8. https://doi.org/10.1038/ncomms3203

4. Roussarie JP, Yao V, Rodriguez-Rodriguez P et al. (2020). Selective Neuronal Vulnerability in Alzheimer's Disease: A Network-Based Analysis. *Neuron*. https://doi.org/10.1016/j.neuron.2020.06.010
