# Supplementary material for: A cell autonomous regulator of neuronal excitability modulates tau in Alzheimer’s disease vulnerable neurons
Source: Brain. 2024 Mar 11;147(7):2384–99. doi: 10.1093/brain/awae051 (PMC11224620; doi:10.1093/brain/awae051)
Supplement: awae051_Supplementary_Data [file awae051_supplementary_data.zip › brain-2023-02150-File009.pdf]

Supplementary material

Supplementary Methods.

Supplementary Figure 1. *DEK* expression in the mouse and the human brain. (A) Scatter plots of *DEK* expression levels in different cell types of the mouse brain. **(B)** Scatter plots of *DEK* expression levels in different cell types of the human brain. Source: Allen brain map transcriptomics explorer.

Supplementary Figure 2. Experimental design for *Dek* silencing in ECII bacTRAP mouse and gene expression changes in EC neurons triggered by DEK overexpression *in vitro.* (A) Schematic representation of the experimental design for the analysis of the effect of *Dek* silencing in ECII neurons in *vivo* by bacTRAP-RNAseq. **(B)** Heatmap of the DEGs between control and DEK-overexpressing primary neurons. **(C)** Volcano plot of DEGs between control and DEK-overexpressing primary neurons. **(D)** Pathways that are altered in DEK-overexpressing primary neurons compared to control.

Supplementary Figure 3. Significant enrichment of genes changing *in vitro* from *Dek* silencing in *in silico* ECII network predictions. Enrichment analysis of DEGs (FDR<0.05, denoted in the rug plot, bottom) between control and *Dek*-silenced ECII neurons *in vitro* demonstrates strong enrichment within genes ranked by probability of functional interaction with *DEK* in the ECII network (blue). Dashed line represents expected enrichment if given random predictions.

Supplementary Figure 4. Effect of *Dek* silencing in EC neurons *in vitro*. (A) Heatmap of the differentially expressed potassium and calcium channel subunits between control and *Dek*-silenced EC primary neurons (n = 3 wells for each group; padj < 0.001). **(B)** Western blot analysis of the levels of total DEK (green) in EC neurons in primary culture at 4-days post-transduction (one representative experiment is shown with n = 3 replicates). The graphs show protein levels in % relative to control neurons. Band intensity was normalized with GAPDH. Unpaired t-test p-value < 0.0001. **(C-E)** Western blot analysis of the levels of phosphor-tau Ser202/Thr205 (C), Thr231 (D) and Thr181 (E) in EC neurons in primary culture at 4-days post-transduction (one representative experiment is shown with n = 2 replicates per group). The graphs show protein levels in % relative to control neurons. Band intensity

was normalized with total tau. Unpaired t-test p-value = 0.166 (C), 0.137 (D) and 0.0025 (E), (2 independent experiments with 3 replicates per group).

Supplementary Figure 5. Effect of *Dek* silencing in ECII neurons and microglia of hMAPT mouse. **(A)** Schematic representation of the experimental design for the time-course analysis of the effect of *Dek* silencing in hMAPT mice. **(B)** Confocal microscopy images of immunofluorescence staining of transduced neurons (mCherry, magenta), ECII neurons (Reelin, cyan) and phospho-tau Thr231 (AT180, yellow) at 1-week post-transduction of control or *Dek*-silencing AAVs in hMAPT mice. Scale bar 100 µm. **(C)** Confocal microscopy images of immunofluorescence staining of *Dek*-silencing AAVs-transduced neurons (mCherry, magenta) and microglia (Iba1, yellow) at 2 weeks post-transduction. **(D-I)** RT-qPCR quantification of the expression levels of *Cx3cr1* (A), *C1qa* (B), *Itgam* (C), *Tyrobp* (D), *Spp1* (E) and *Ccl3* (F) in bulk homogenates of the control and the *Dek*-silenced opposite hemisphere for each hMAPT mouse fed with control diet at 2 weeks post-transduction. Paired t-test p-value = 0.0017 (A), 0.023 (B), 0.0098 (C), 0.0002 (D), 0.047 (E), 0.0023 (F).

Supplementary Figure 6. Effect of *Dek* silencing in ECII neurons of control and PLX5622 diet treated hMAPT mouse. **(A)** Schematic representation of the experimental design for the analysis of the effect of *Dek* silencing in hMAPT mice fed with control diet or PLX5622 containing diet at 2 weeks post-transduction. **(B)** Western blot analysis of Iba1 levels (red) in control or PLX5622 diet fed mice and, in the control, or the *Dek*-silenced hemisphere of the same representative mice. The graph shows the quantification for the complete mouse cohort, paired by the control and the *Dek*-silenced opposite hemisphere for each mouse. Iba1 levels were normalized by actin. 2way ANOVA Sidak's multiple comparisons test p-value = 0.023 (control side, control diet vs. *Dek*-silencing side, PLX5622 diet), 0.0064 (*Dek*-silencing side control vs. PLX5622 diet). **(C)** Confocal microscopy images of immunofluorescence staining of transduced neurons (mCherry, magenta), ECII neurons (Reelin, cyan) and microglia (Iba1, yellow) at 2 weeks post-transduction of control or PLX5622 diet fed hMAPT mice. Scale bar 100 µm. The graphs show the quantification of Iba1 fluorescence intensity (upper panel) and the number of reelin positive cells (lower panel), paired by the control and the *Dek*-silenced opposite hemisphere for each hMAPT mouse. 2way ANOVA Sidak's multiple comparisons test Iba1 p-value = 0.0130 (control side, control vs. PLX5622 diet), 0.0060 (*Dek*-silencing side, control vs. PLX5622 diet); Reelin p-value = 0.0105 (control diet, control vs. *Dek*-silencing side).

Supplementary Figure 7. Schematic representation of the effect of *Dek* silencing on EC neuron function and tau pathology. *Dek* silencing causes tau (red pies) accumulation in neurons likely via impairment of the autophagy flux (autophagosomes in blue, lysosomes in red). It also causes epigenetic changes across genome, in particular an increase of the histone mark H3.1K36 acetylation (orange circles). Chromatin accessibility is increased around the *Egr1* locus, which unleashes *Egr1* upregulation upon membrane depolarization (turquoise receptors). A number of electrophysiological properties of the cell are altered, making ECII neurons more excitable. In culture, these changes are compensated for by a decrease in expression of all AMPA receptor subunits, and eventually lower baseline expression of immediate early genes. Lastly, some of these neuronal changes cause reactivity of microglia (salmon cells) *in vivo*, eventually leading to ECII neuron degeneration. Created with BioRender.com.

Supplementary Table 1. Top 20 hits with highest connectivity scores to the NetWAS 2.0 vulnerability module and their respective associations with MAPT in the ECII neuron network.

Supplementary Table 2. Effects of the Dek silencing and overexpression. Genes that are differentially expressed between *Dek*-silenced and control neurons *in vitro* (1) and *in vivo* (2), and between neurons where *Dek* was overexpressed and control neurons *in vitro* (3). Results from the ATACseq (4), Mod Spec (5) and CUT&Tag (6) experiments. Results from the mass spectrometry analysis of protein abundance in *Dek*-silenced and control EC primary neurons (7). DeepSEA predictions for rs145578678 and SNPs in LD with it in populations with European ancestry. Predictions were filtered for p-value < 0.05 after Bonferroni correction were considered, and for observations specific to brain (8).

Supplementary Table 3. Electrophysiological properties describing active, passive, and excitatory input in control and *Dek*-silenced neurons. Bold values indicate statistical significance.

Figure S1

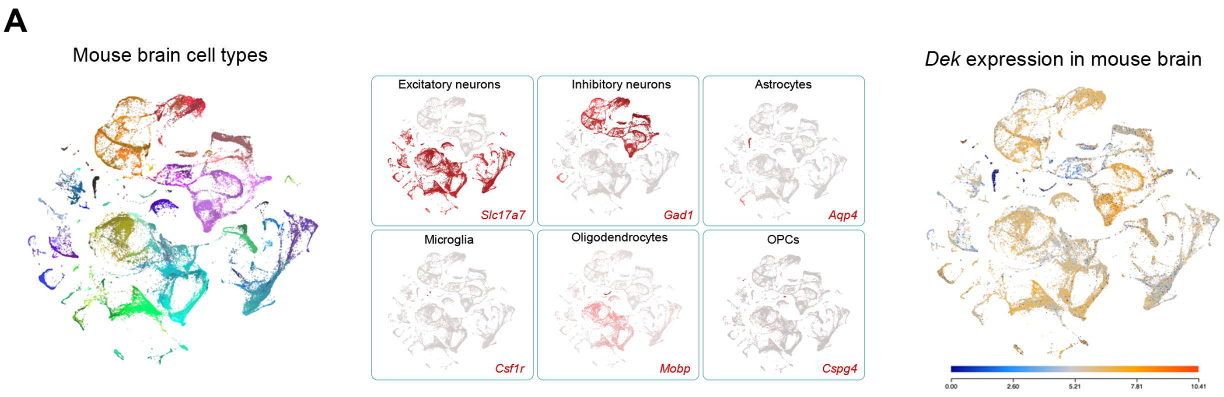
A
Mouse brain cell types
Excitatory neurons
Slc17a7
Inhibitory neurons
Gad1
Astrocytes
Aqp4
Microglia
Csf1r
Oligodendrocytes
Mobp
OPCs
Cspg4
Dek expression in mouse brain
0.00
2.60
5.21
7.81
10.41

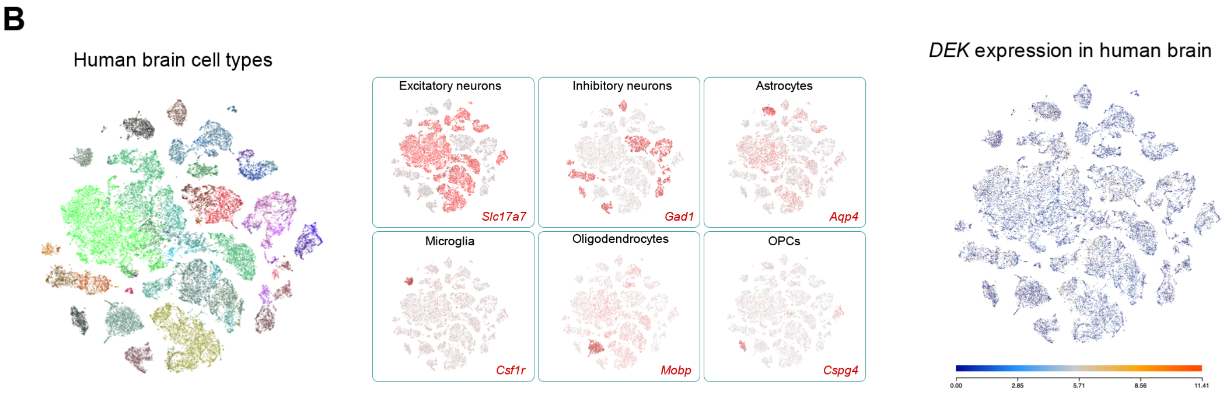
B
Human brain cell types
Excitatory neurons
Slc17a7
Inhibitory neurons
Gad1
Astrocytes
Aqp4
Microglia
Csf1r
Oligodendrocytes
Mobp
OPCs
Cspg4
DEK expression in human brain
0.00
2.85
5.71
8.56
11.41

Figure S2

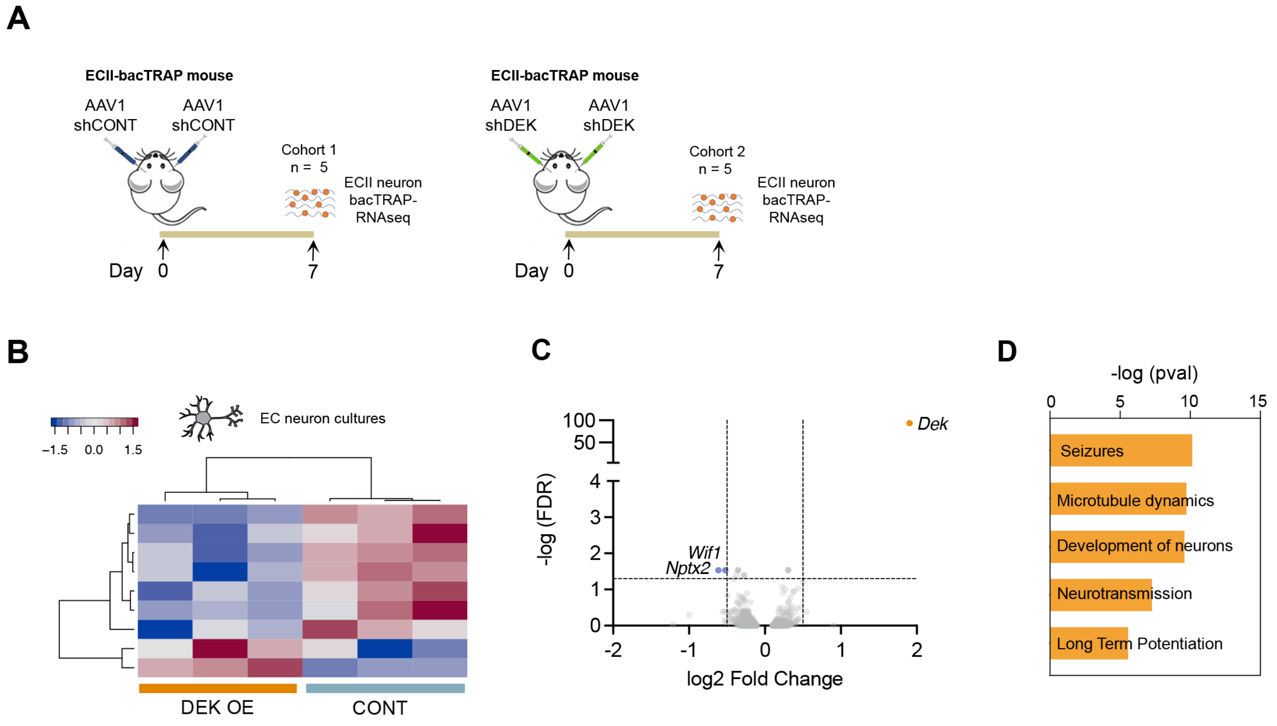
A
ECII-bacTRAP mouse
AAV1
shCONT
AAV1
shCONT
Cohort 1
n = 5
ECII neuron
bacTRAP-
RNAseq
Day 0
7
ECII-bacTRAP mouse
AAV1
shDEK
AAV1
shDEK
Cohort 2
n = 5
ECII neuron
bacTRAP-
RNAseq
Day 0
7
B
−1.5 0.0 1.5
EC neuron cultures
DEK OE
CONT
C
Dek
Wif1
Nptx2
-log (FDR)
100
50
4
3
2
1
0
-2 -1 0 1 2
log2 Fold Change
D
-log (pval)
0 5 10 15
Seizures
Microtubule dynamics
Development of neurons
Neurotransmission
Long Term Potentiation

A

Figure S3

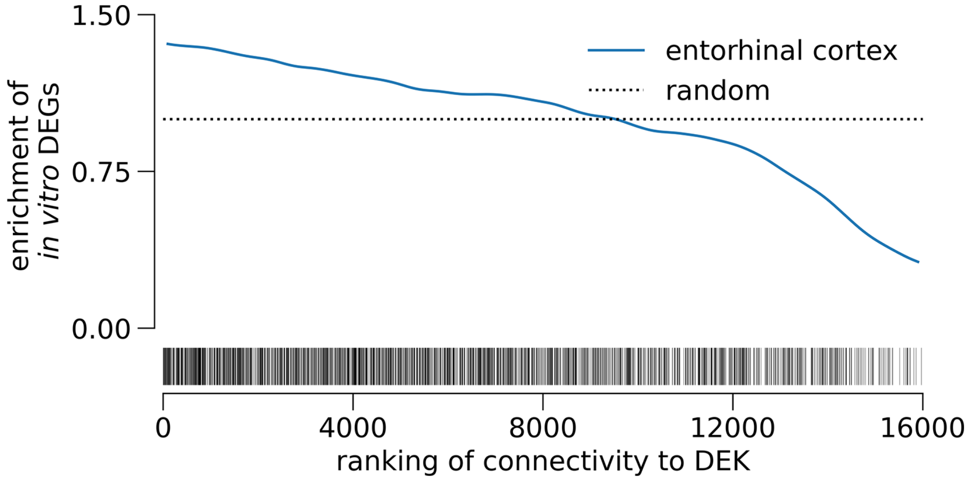

1.50
0.75
0.00
enrichment of
in vitro DEGs
entorhinal cortex
random
0
4000
8000
12000
16000
ranking of connectivity to DEK

Figure S4

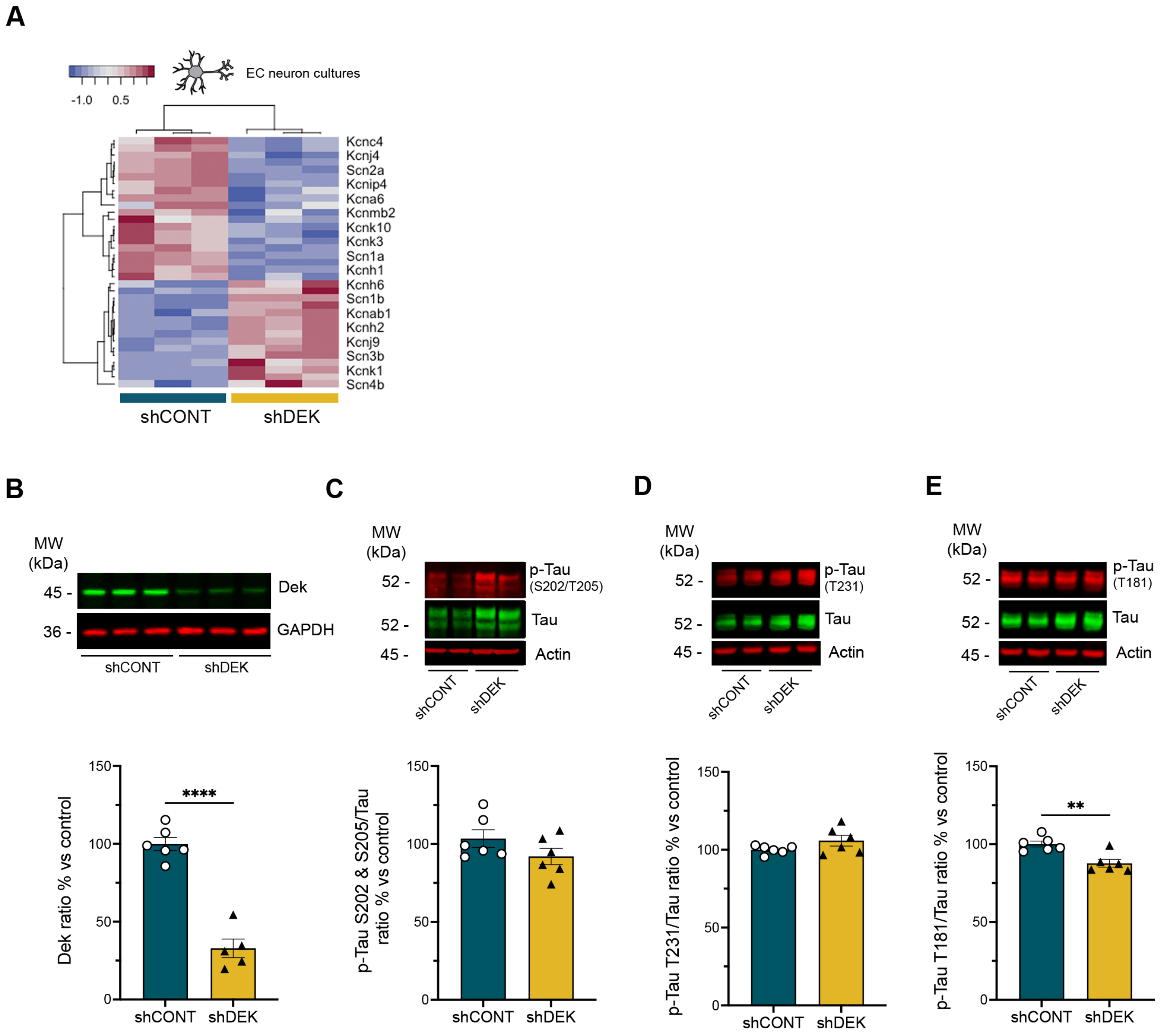
A
-1.0
0.5
EC neuron cultures
Kcnc4
Kcnj4
Scn2a
Kcnip4
Kcna6
Kcnmb2
Kcnk10
Kcnk3
Scn1a
Kcnh1
Kcnh6
Scn1b
Kcnab1
Kcnh2
Kcnj9
Scn3b
Kcnk1
Scn4b
shCONT
shDEK
B
MW (kDa)
45 -
36 -
Dek
GAPDH
shCONT
shDEK
Dek ratio % vs control
****
C
MW (kDa)
52 -
52 -
45 -
p-Tau (S202/T205)
Tau
Actin
p-Tau S202 & S205/Tau ratio % vs control
D
p-Tau (T231)
p-Tau T231/Tau ratio % vs control
E
p-Tau (T181)
p-Tau T181/Tau ratio % vs control
**
150
100
50
0

Figure S5

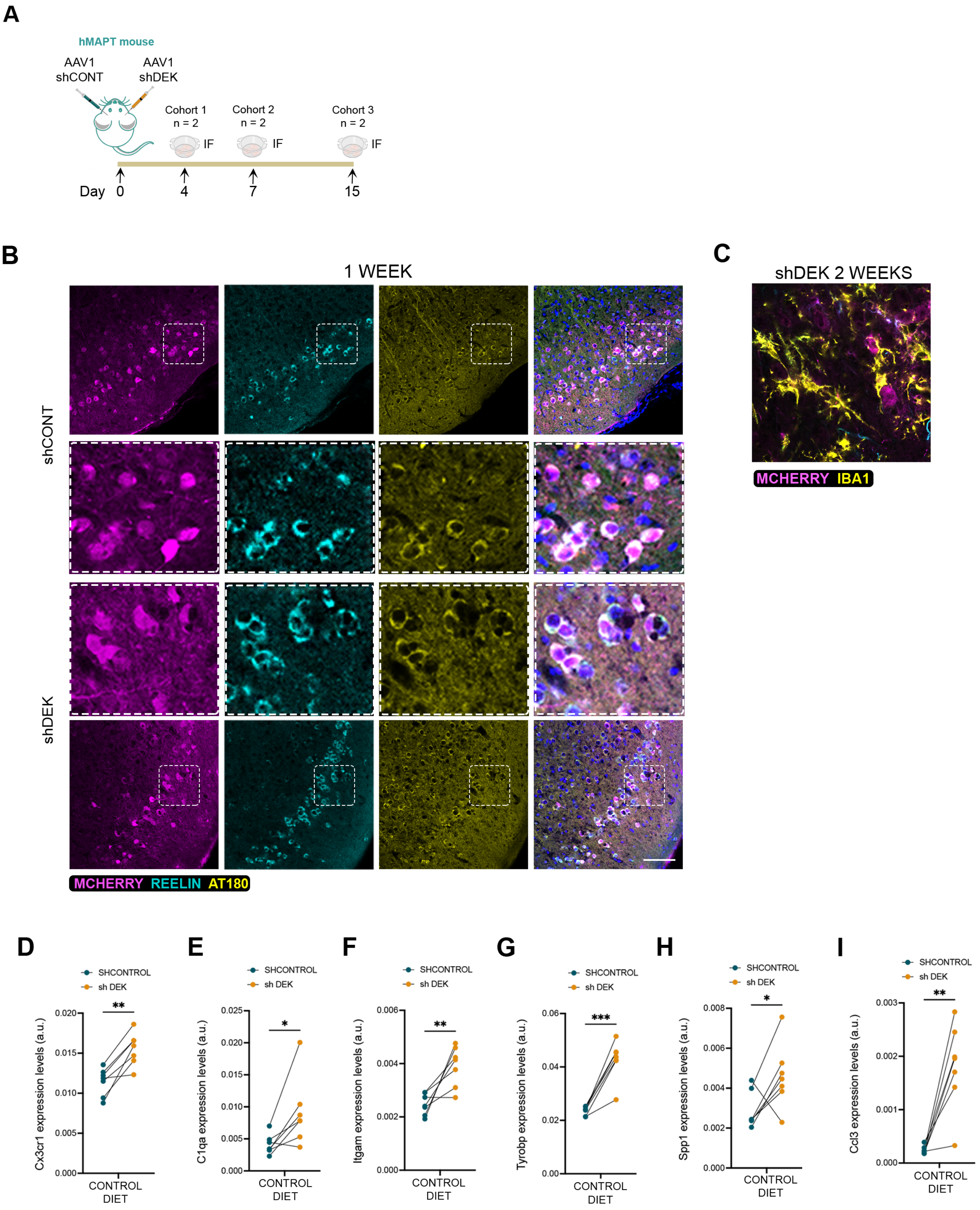

A
hMAPT mouse
AAV1 shCONT
AAV1 shDEK
Cohort 1 n = 2
Cohort 2 n = 2
Cohort 3 n = 2
IF
Day 0 4 7 15
B
1 WEEK
shCONT
shDEK
MCHERRY REELIN AT180
C
shDEK 2 WEEKS
MCHERRY IBA1
D
SHCONTROL
sh DEK
Cx3cr1 expression levels (a.u.)
0.000 0.005 0.010 0.015 0.020
**
CONTROL DIET
E
SHCONTROL
sh DEK
C1qa expression levels (a.u.)
0.000 0.005 0.010 0.015 0.020 0.025
*
CONTROL DIET
F
SHCONTROL
sh DEK
Itgam expression levels (a.u.)
0.000 0.002 0.004 0.006
**
CONTROL DIET
G
SHCONTROL
sh DEK
Tyrobp expression levels (a.u.)
0.00 0.02 0.04 0.06
***
CONTROL DIET
H
SHCONTROL
sh DEK
Spp1 expression levels (a.u.)
0.000 0.002 0.004 0.006 0.008
*
CONTROL DIET
I
SHCONTROL
sh DEK
Ccl3 expression levels (a.u.)
0.000 0.001 0.002 0.003
**
CONTROL DIET

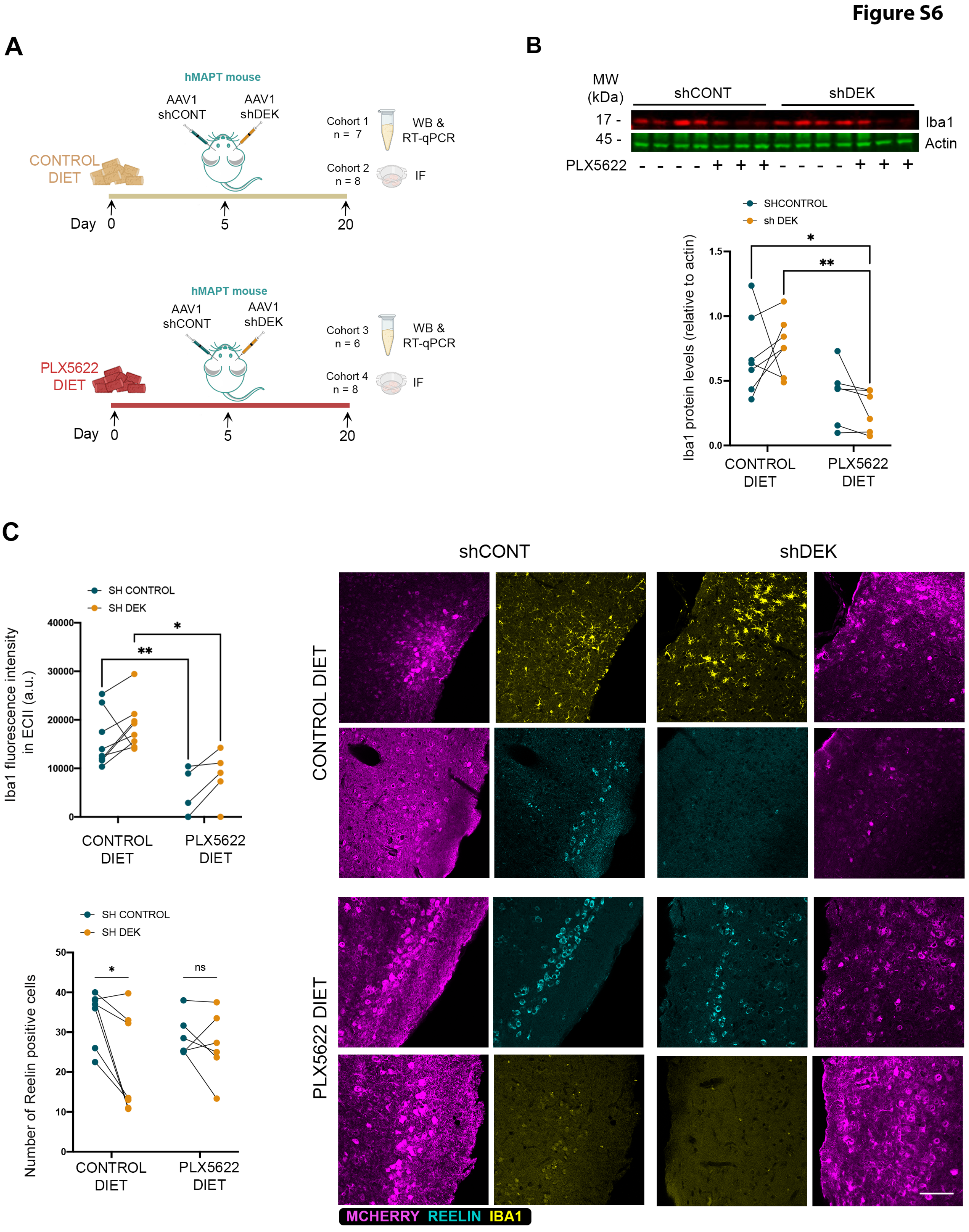
Figure S6
A
hMAPT mouse
AAV1 shCONT
AAV1 shDEK
CONTROL DIET
Cohort 1 n = 7
WB & RT-qPCR
Cohort 2 n = 8
IF
Day 0 5 20
hMAPT mouse
AAV1 shCONT
AAV1 shDEK
PLX5622 DIET
Cohort 3 n = 6
WB & RT-qPCR
Cohort 4 n = 8
IF
Day 0 5 20
B
MW (kDa)
shCONT
shDEK
17 -
45 -
Iba1
Actin
PLX5622 - - - - + + + - - - - + + +
SHCONTROL
sh DEK
Iba1 protein levels (relative to actin)
CONTROL DIET
PLX5622 DIET
C
SH CONTROL
SH DEK
Iba1 fluorescence intensity in ECII (a.u.)
CONTROL DIET
PLX5622 DIET
SH CONTROL
SH DEK
Number of Reelin positive cells
ns
CONTROL DIET
PLX5622 DIET
shCONT
shDEK
CONTROL DIET
PLX5622 DIET
MCHERRY REELIN IBA1

Figure S7

A

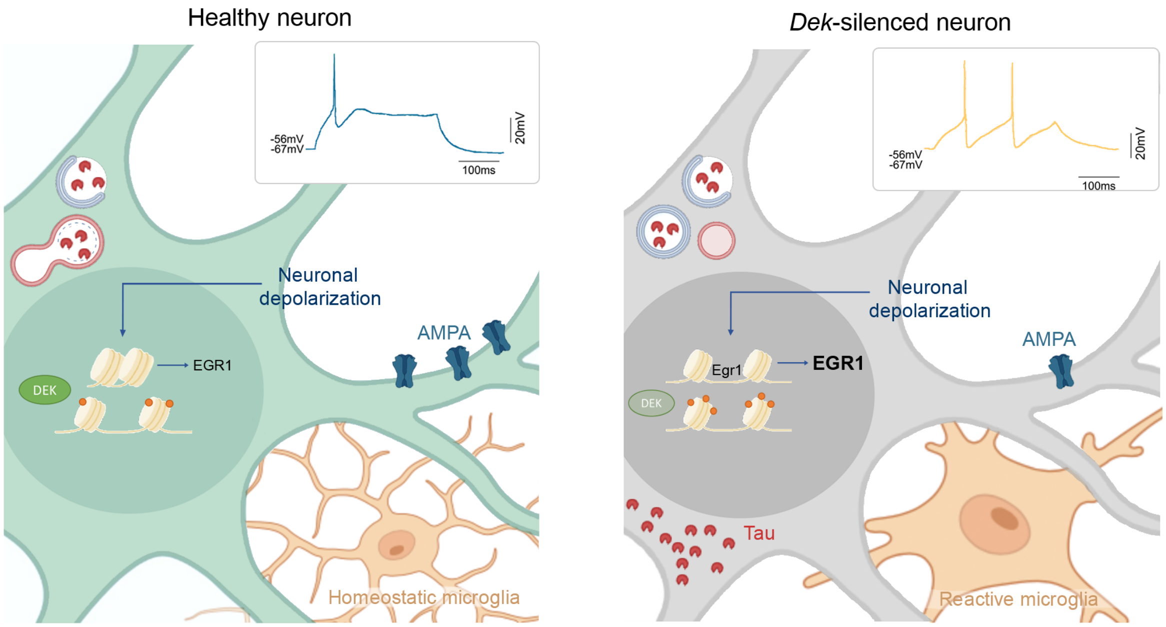

Healthy neuron
-56mV
-67mV
20mV
100ms
Neuronal depolarization
EGR1
DEK
AMPA
Homeostatic microglia
Dek-silenced neuron
-56mV
-67mV
20mV
100ms
Neuronal depolarization
Egr1
EGR1
DEK
AMPA
Tau
Reactive microglia

Table S1

	Connectivity to Netwas2.0 ECII neuron vulnerability module (Roussarie et al, 2020)	Average edge score to MAPT in ECII neuron network alz.princeton.edu
DEK	10.45	0.976
TMX1	9.06	0.948
PRKD3	8.94	0.763
RECQL	8.89	0.87
MOB1A	8.81	0.958
EXOSC8	8.18	0.917
SYPL1	8.18	0.996
SMC4	8.17	0.844
HAT1	8.14	0.954
NDC1	8.13	0.693
CCNA2	8.03	0.654
ANP32E	7.94	0.928
HEATR1	7.94	0.922
U2SURP	7.92	0.961
DIMT1	7.89	0.915
NUP54	7.87	0.932
SNAP23	7.84	0.886
SRSF10	7.73	0.826
PPP1CC	7.72	0.866
NUP107	7.68	0.851

Table S3

Electrophysiological properties	Control (n=10)	shDEK (n=11)	p value
**Passive properties**			
Resting Membrane Potential (mV)	-66.97 ± 6.001	-59.05 ± 8.321	**0.0227**
Membrane Capacitance (pF)	17.56 ± 3.531	16.26 ± 4.573	0.4786
τ (μs)	624.8 ± 124.2	682.6 ± 298.5	0.5944
Input Resistance (MΩ)	159.4 ± 59.17	115.1 ± 24.19	**0.0336**
Sag Amplitude (mV)	2.595 ± 1.747	9.407 ± 9.321	**0.0495**
**Active properties**			
Rheobase			
Current threshold (pA)	60.00 ± 24.13	44.55 ± 22.96	**0.0368**
First-spike Latency (ms)	48.83 ± 17.35	75.53 ± 40.45	0.0824
AP waveform			
Amplitude (mV)	50.38 ± 6.823	54.19 ± 9.196	0.1621
Firing threshold (mV)	-31.20 ± 2.513	-26.34 ± 4.144	**0.0046**
Half-width (ms)	2.662 ± 0.4964	3.158 ± 0.7493	0.0930
Max Rise Slope (mV/ms)	52.46 ± 23.79	35.81 ± 16.75	0.0771
Time to Max Rise Slope (ms)	4.880 ± 0.3743	5.150 ± 0.2	0.0503
Max Decay Slope (mV/ms)	-21.91 ± 6.245	-16.79 ± 6.214	0.0753
Time to Max Decay Slope (ms)	7.320 ± 0.4523	7.750 ± 0.718	0.1265
**Spontaneous Excitatory Input**			
sEPSC amplitude (pA)	26.27 ± 13.44	17.44 ± 6.515	0.0668
sEPSC Frequency (Hz)	2.667 ± 1.706	2.070 ± 0.9253	0.3251
sEPSC Charge Transfer (pC)	17.97 ± 12.79	9.465 ± 6.833	0.1971
