# Supplementary figures and images for: A cell autonomous regulator of neuronal excitability modulates tau in Alzheimer’s disease vulnerable neurons
Source: Brain. 2024 Mar 11;147(7):2384–99. doi: 10.1093/brain/awae051 (PMC11224620; doi:10.1093/brain/awae051)

Figure 4A and S4C

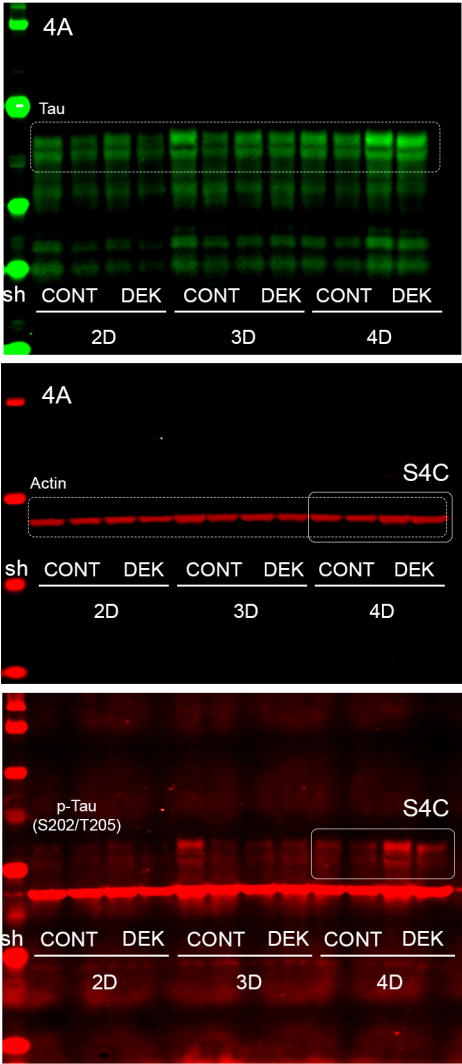

Figure 4D

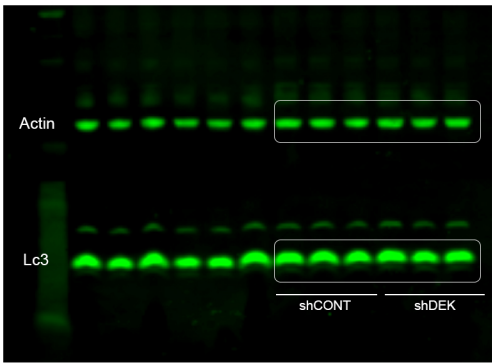

Figure S4B

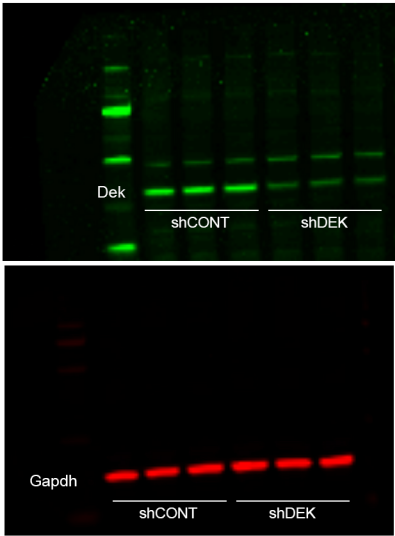

Figure S4D

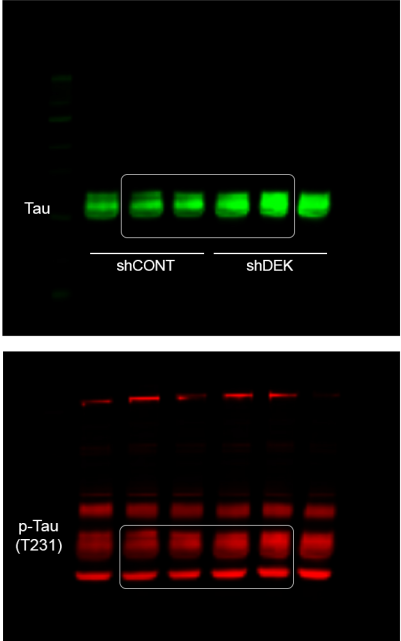

Figure S4E

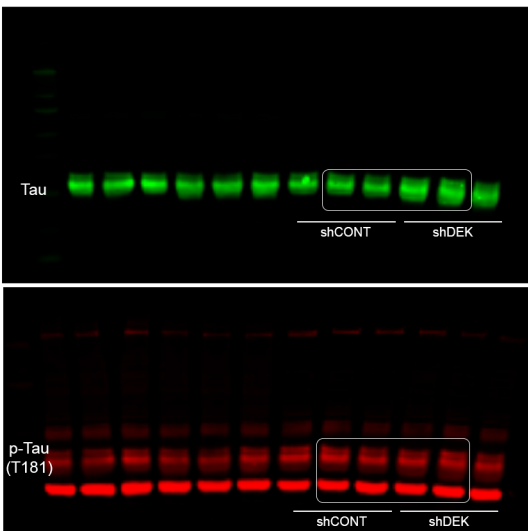

Figure S6B

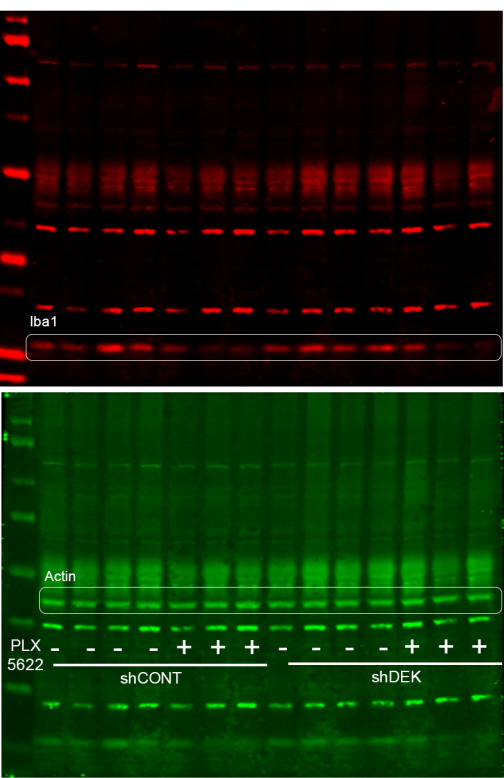

Supplement: awae051_Supplementary_Data [file awae051_supplementary_data.zip › brain-2023-02150-File010.pdf]
